# Low-Intensity Focused Ultrasound Stimulation Ameliorates Working Memory Dysfunctions in Vascular Dementia Rats *via* Improving Neuronal Environment

**DOI:** 10.3389/fnagi.2022.814560

**Published:** 2022-02-21

**Authors:** Faqi Wang, Qian Wang, Ling Wang, Jing Ren, Xizi Song, Yutao Tian, Chenguang Zheng, Jiajia Yang, Dong Ming

**Affiliations:** ^1^Academy of Medical Engineering and Translational Medicine, Tianjin University, Tianjin, China; ^2^College of Precision Instruments and Optoelectronics Engineering, Tianjin University, Tianjin, China; ^3^Tianjin Key Laboratory of Brain Science and Neuroengineering, Tianjin, China

**Keywords:** low intensity focused ultrasound, vascular dementia, working memory, cerebral blood flow, synaptic function, neuroinflammation

## Abstract

Working memory impairment is one of the remarkable cognitive dysfunctions induced by vascular dementia (VD), and it is necessary to explore an effective treatment. Recently, low-intensity focused ultrasound stimulation (LIFUS) has been found notable neuroprotective effects on some neurological diseases, including VD. However, whether it could ameliorate VD-induced working memory impairment was still not been clarified. The purpose of this study was to address this issue and the underlying mechanism. We established VD rat model using the bilateral common carotid artery occlusion (BCCAO) and applied the LIFUS (center frequency = 0.5 MHz; I_spta_ = 500 mW/cm^2^, 10 mins/day) to bilateral medial prefrontal cortex (mPFC) for 2 weeks since 2 weeks after the surgery. The main results showed that the LIFUS could significantly improve the performance of VD rats in the specific working memory tasks (delayed nonmatch-to-sample task and step-down task), which might be associated with the improved synaptic function. We also found the improvement in the cerebral blood flow (CBF) and reduced neuroinflammation in mPFC after LIFUS treatment indicated by the inhibition of Toll-like receptor (TLR4)/nuclear factor kappa B (NF-κB) pathway and the decrease of proinflammatory cytokines. The amelioration of CBF and neuroinflammation may promote the living environment of the neurons in VD which then contribute to the survival of neurons and the improvement in synaptic function. Taken together, our findings indicate that LIFUS targeted mPFC can effectively ameliorate reward-based spatial working memory and fear working memory dysfunctions induced by VD *via* restoring the living environment, survivability, and synaptic functions of the neurons in mPFC of VD rats. This study adds to the evidence that LIFUS could become a promising and non-invasive treatment strategy for the clinical treatment of central nervous system diseases related to cognitive impairments in the future.

## Introduction

Dementia is a common public health problem in the world with alarming increases in the prevalence. Additionally, the vascular dementia (VD) is a very frequent form of dementia only after Alzheimer’s disease (AD). The prevalence rises with age, with a risk of VD roughly doubling every 5.3 years (O’Brien and Thomas, 2015). However, unlike AD, no curative treatment is yet available for VD in clinic at present (O’Brien and Thomas, 2015; Eguchi et al., 2018). VD is a progressive disease caused by long-term chronic cerebral hypoperfusion (CCH). Cerebral ischemia is a critical cause of neuronal loss and synaptic disintegration. Recent evidence suggests that CCH is associated with systemic inflammation before neurological symptoms develop in VD (Poh et al., 2020). Neuroinflammation caused by cerebral ischemia could aggravate the damage of neurons (Wang et al., 2020). Both in clinical and in basic research, it has been found that VD affects many cognitive abilities including working memory (Venkat et al., 2015). Working memory is a crucial component of memory processes, and it is necessary for higher cognitive and executive functions, which include comprehension, language, learning, reasoning, and thinking (Baddeley, 2010; Riley and Constantinidis, 2015). Although it has been shown that working memory impairment is one of the remarkable symptoms after VD (Blom et al., 2019), there is still a lack of effective treatment methods for working memory impairment caused by VD. Therefore, there is an urgent need for novel and effective treatment strategies.

The prefrontal cortex (PFC) plays a critical role for resilient information maintenance during whole performing working memory tasks (Eriksson et al., 2015). In addition, the medial prefrontal cortex (mPFC), a subregion of PFC, is essential for cognitive process including working memory (Euston et al., 2012; Xu et al., 2019). Prior studies have found that the mPFC plays an important role in a variety of working memory processes, which include spatial working memory (Toepper et al., 2014; Hallock et al., 2016; Tamura et al., 2017), emotional working memory (Smith et al., 2018; Manelis et al., 2020), olfactory working memory (Liu et al., 2014; Nguyen et al., 2020), and tactile working memory (Esmaeili and Diamond, 2019). Therefore, mPFC may become a promising target for improving the working memory impairments caused by VD.

Non-invasive brain stimulation technologies have gradually played an important role in the researches of neuroscience and neurological diseases in recent decades. Low-intensity focused ultrasound stimulation (LIFUS), as an emerging non-invasive neuromodulation, has been rapidly developed with the advantage of excellent targeting, penetration depth, and spatial resolution (Yuan et al., 2021). Among previous animal reports, LIFUS has been proved to be useful in treating various neurological and psychiatric diseases such as ischemic brain injury (Guo et al., 2015; Li et al., 2017), depression (Zhang et al., 2019, 2021), and dementia (Huang et al., 2017; Eguchi et al., 2018; Bobola et al., 2020). Although low-intensity ultrasound therapy has been used to modulate cognitive impairments induced by VD, whether LIFUS targeting the mPFC could ameliorate the working memory impairment and the underlying mechanisms are still unknown. A series of previous studies have found that LIFUS could promote cerebral angiogenesis in rodents (Huang et al., 2017; Li et al., 2017; Eguchi et al., 2018). Meanwhile, chronic cerebral ischemia is one of the main pathogenic factors of VD. Therefore, promoting cerebral angiogenesis may be one of the mechanisms for LIFUS treating VD. Among several studies on therapeutic applications of LIFUS, it has been found that ultrasound stimulation can suppress the neuroinflammation induced by lipopolysaccharide (LPS) both *in vivo* and *in vitro* by modulation of Toll-like receptor (TLR4)/nuclear factor kappa B (NF-κB) pathway (Liu et al., 2017; Chen et al., 2019; Chang et al., 2020). Therefore, we hypothesized that the antiinflammatory effect of LIFUS may be one of the potential mechanisms for ameliorating the cognitive impairments induced by VD.

Thus, this study aims to explore the effects of LIFUS on working memory impairments induced by VD and the underlying mechanisms. Here, we first showed that LIFUS targeted mPFC could restore specific working memory dysfunctions induced by VD. Additionally, it could improve Nissl bodies expression and synaptic functions in mPFC of VD rats. Furthermore, the underlying mechanism may be that LIFUS could increase the CBF and inhibit the TLR4/NF-κB pathway. Overall, our findings reveal that the LIFUS targeted mPFC may be a potential therapeutic method in central nervous system diseases related to cognitive impairments.

## Materials and Methods

### Animals

Twenty-five adult male Wistar rats (170–210 g) were purchased from Beijing Vital River Laboratory Animal Technology Co. Before the experiment, animals were housed five per cage and allowed to acclimate for 1 week. The house environment was controlled at 23°C ± 2°C and 5060% humidity with a 12-h light–dark cycle (with lights on and off at 20:00 and 8:00, respectively). Unless otherwise specified, rats could receive food and water *ad libitum*. All experiments’ procedures were carried out following the Animal Management Rules of the Ministry of Health of the People’s Republic of China and approved by the Animal Research Ethics Committee of Tianjin Hospital.

### Vascular Dementia Model

The VD model was established by typical bilateral common carotid artery occlusion (BCCAO). Rats were fasted but free to water, 6 h before the surgery. Following that, rats were anesthetized using 10% chloral hydrate (350 mg/kg, i.p., Beijing Dingguo Changsheng Biotechnology Co., Ltd., Beijing, China). After anesthesia, atropine (0.09 ml/kg, i.p., Shanghai full woo Biotechnology (Zhumadian)Co., Ltd., Shanghai, China) was used to inhibit salivary secretion. Then, the VD rats underwent the BCCAO surgery as previously published (Yang et al., 2017). First, a ventral midline incision was made in the neck and the muscle retracted on either side of the trachea to expose both the right and left common carotid arteries, around which loose threads were placed. Second, the vessel was fully ligated, and the wound was sutured. During the surgery, the rats were maintained normal respiratory tract and body temperature with a homeothermic monitoring system (RWD, Shenzhen, China). After the surgery, rats were injected flurbiprofen axetil (0.45 ml/kg, i.p., Beijing Tide Pharmaceutical Co., Ltd., Beijing, China) for postoperative analgesia. After coming to their senses, all rats were reared in the animal house and given free access to food and water.

### Low-Intensity Focused Ultrasound Stimulation Procedure

**FIGURE 1:**
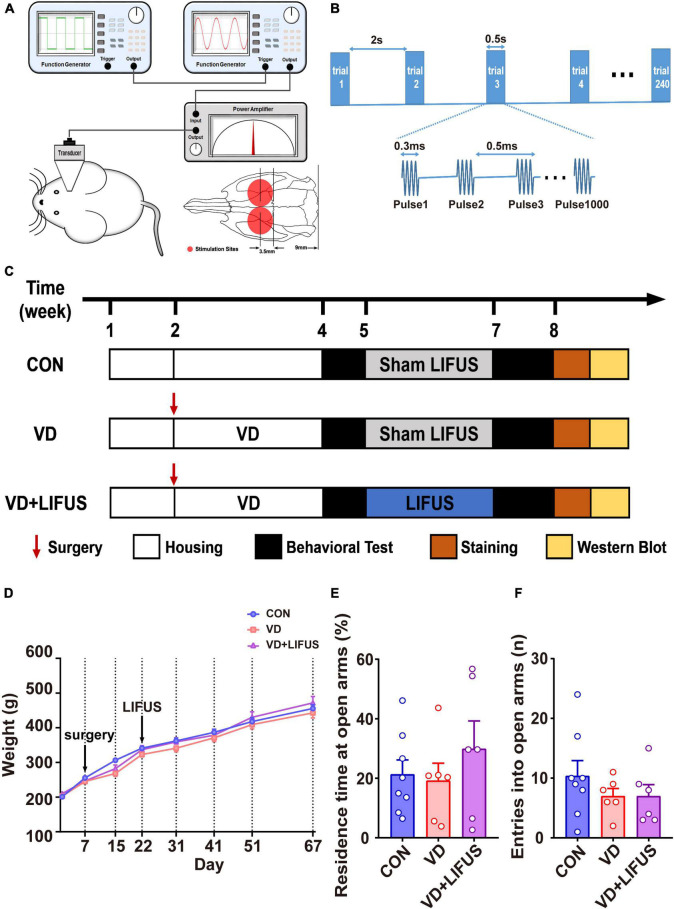
**(A)** Illustration of LIFUS system. **(B)** Illustration of LIFUS parameters. **(C)** The timeline of the whole experiment. **(D)** Changes in body weight during the experiment (CON, *n* = 8; VD, *n* = 6; VD + LIFUS, *n* = 6). **(E,F)** The residence time at open arms **(E)** and entries into open arms in the OPT (CON, *n* = 8; VD, *n* = 6; VD + LIFUS, *n* = 6).

The LIFUS system consists of (1) two function generators (DG4162 and DG822, RIGOL, Beijing, China), (2) a custom-designed radio frequency amplifier (SWA400A, North Star, Shijia zhuang, China), (3) a 0.5MHz single element immersion transducer (V318, Olympus, Tokyo, Japan), and (4) a custom-designed acoustic collimator (Zhang et al., 2019). The schematic diagram of LIFUS system is shown in Figure 1A. The LIFUS parameters used in this study were as follows (shown in Figure 1B): center frequency = 0.5 MHz; pulse repetition frequency (PRF) = 2.0 kHz; the number of cycles = 150 (0.3 ms tone burst duration, TBD); the sonication duration (SD) = 0.5 s; the interstimulus interval (ISI) = 2 s, and the spatial peak temporal average intensity (I_spta_) = 500 mW/cm^2^. The targets of LIFUS are the bilateral mPFC. In addition, the LIFUS treatment was performed daily for 10 min on each side. During the LIFUS, all rats were mounted on the stereotaxic apparatus (RWD, Shenzhen, China) and anesthetized with 1% isoflurane (RWD, Shenzhen, China). Before applying the LIFUS, the hair on the bilateral mPFC was shaved. The other rats in either CON or VD groups underwent the same procedures including anesthesia but without LIFUS.

### Experimental Procedure

First, twenty-five rats were randomly divided into control group (CON, *n* = 8) and VD model group (*n* = 17). The rats in VD model group received the BCCAO surgery and recovered for 2 weeks. Due to the survival rate of surgery being about 70%, twelve rats of the VD model group survived. Then, the survival rats were randomly divided into two subgroups, VD group (VD model rats without LIFUS, *n* = 6) and VD + LIFUS group (VD model rats with LIFUS treatment, *n* = 6). VD + LIFUS group was treated by LIFUS daily for 14 days starting after 2 weeks’ recovery whereas CON and VD groups with sham LIFUS. At the end of the LIFUS, the behavioral tests were performed to evaluate the effects of LIFUS on working memory. After all behavioral tests, the cerebral blood flow (CBF) was measured to examine the change of ischemia. In addition, the histological and western blotting analyses were performed to explore the underlying mechanisms. The experimental schedule is shown in Figure 1C.

### Behavioral Tests

All behavioral tests were carried out in a dimly lit and quiet room and during the dark phase of the light–dark cycle. In every behavioral test, the equipment and the objects used were cleaned with 75% ethanol solution and water in order between each trial to eliminate odor cues.

#### Elevated-Plus Maze Task

A standard elevated-plus maze was used to assess the anxiety-associated behavior. When starting the test, rats were placed into the center square facing a same open arm and allowed to explore freely for 5 min. The activity track of each rat was recorded by Smart video tracking software (Panlab, Holliston, United States). The evaluation indexes were the number of entries into open arms and the percentage of residence time in the open arms.

#### Delayed Nonmatch-to-Sample Task

This test was performed to evaluate spatial working memory based on a reward using a standard Y maze equipment. The delayed nonmatch-to-sample task includes three periods: (1) adaptation period, (2) training period, and (3) testing period. In the 4 days’ adaptation period, all groups were restricted drinking water and could drink water for 2 h daily to maintain their water desire. Before the training period, rats underwent 3 days of habituation in the Y maze that they could visit three arms (all with 150 μl water) freely for 10 min. In the 2-day training period, only two arms (baited arms) were placed 150 μl water. The rats were forced to explore each baited arm in turn 30 min daily for reward. During the 4-day testing period, each rat underwent 10 trials per day. Each trial contained three stages. First, the rat was placed into the starting arm and forced into one of the baited arms randomly obtaining a 150 μl water reward (sample stage). Next, the rat returned the starting arm for a thirty-s delay stage. Then, both baited arms were accessible for the rat during the choice stage. But the rat could get a 150 μl water reward only when it chose the baited arm which was different from that during the sample stage and this trial was recorded as “correct.” The rat was immediately guided back to the starting arm waiting for next trial whether this trial was correct or not. The interval between the two trials was 60 s. The evaluation indexes were the correct rate and reaction time. The reaction time was defined as the time spent from start to choice point (30 cm away from the end of baited arms).

#### Novel Object Recognition Test

This test was performed to evaluate working memory of objects based on natural proclivity for exploring novelty. The task contained three periods: (1) habituation period, (2) familiarization period, and (3) testing period. During the habituation periods, the rats were allowed to explore the empty experimental box (60 cm × 60 cm) freely for habituation for 8 min. After 24 h, the rats were allowed to freely explore the box for 5 min with two identical objects placed in opposite corners during the familiarization period. One h later, the rats were placed in the same box again for the test period. During the test period, the rats were also allowed to freely explore the box for 5 min but one of the two familiar objects in the familiarization period was replaced with a novel object. Define exploration as touching or sniffing the object with the forepaws and/or nose. The exploration time of the familiar object (TF) and the novel object (TN) during the testing period was recorded. The discrimination ratio was calculated as TN/(TN + TF).

#### Step-Down Task

This test was performed to evaluate fear working memory based on the fear environment using a step-down reaction box. The bottom of the step-down reaction box is made of parallel stainless-steel bars that can apply electrical stimulation to create a fearful environment for rats. In addition, an insulated platform is placed at the corner of the box. The task also includes three periods: (1) adaptation period, (2) training period, and (3) testing period. In the adaptation period, the rats were allowed to explore the step-down reaction box freely for 5 min without electrical stimulation. The training period was performed after 24 h. During the training period, the rats were placed on the platform in the reaction box. When the rats jumped off the platform, a 0.4 mA electrical stimulation would be applied continuously at an interval of 2 s until the rat jumps on the platform. If one rat could not jump on the platform within 90 s, this rat would be regarded as failed. After 90 min, the rats were placed on the platform again without electrical stimulation. The duration time of test period was 3 min, and the latency of rats jumping off the platform was recorded. Define jumping off the platform as all four claws leaving the platform. The evaluation indexes were the latency to step-down, time on the platform, and the number of down the platform.

### Laser Speckle Contrast Imaging

At the end of all behavioral tests, the laser speckle contrast imaging (LSCI) was carried out to measure CBF by a commercial laser speckle blood flow imager (RFLSI Pro, RWD, Shenzhen, China). To measure the CBF, the rats were mounted on the stereotaxic apparatus (RWD, Shenzhen, China) under anesthesia with 1% isoflurane. The scalp was cut along the midline to expose the skull after shaving the hair. The tissues on the surface of skull were cleaned carefully (Li et al., 2017). Then, using a skull drill (RWD, Shenzhen, China) slowly thinned a 6 mm × 10 mm rectangular cranial window (centered at 1 mm posterior to the bregma). The skull was thinned until the cortical blood vessels were clearly visible. During the whole process, the normal saline was used to cool the skull avoiding brain tissue damaged by excessive temperature. Then, the position of the skull window was placed under the laser speckle imager for laser speckle imaging. The evaluation indexes were the number of vascular branches and the blood flow of the main vein.

### Histological Analysis

In the histological analysis, hematoxylin–eosin staining (HE) was used to detect the morphological changes of neurons in PFC of rats (each group, *n* = 2). The Nissl staining was used to detect the density of Nissl body in rats PFC (each group, *n* = 2). The changes of morphology and density of dendritic spines were measured by the Golgi-cox staining (CON, *n* = 6; VD, *n* = 4; VD + LIFUS, *n* = 4). After the LSCI, all the rats were sacrificed with urethane and perfused with 0.1 M phosphate buffer saline (PBS, pH = 7.4) immediately. For Golgi-cox staining, the brains of rats were removed into the Golgi-cox solution right now. After 12 days, the PFC was sectioned into 150-μm thick slices in coronal plane with an oscillating microtome (Leica VT1200S, Germany). In addition, spine counting was carried out in a single dendrite in each neuron. The dendritic spines were classified into four different types: thin, mushroom, stubby, and branched. The total density of dendritic spines was expressed as the mean of dendritic spines in per unit length. On the other hand, the quantification of dendritic spines according to shape was expressed as a ratio in relation to total dendritic spines (i.e., number of spines of a given shape/total number of spines in each dendrite) (Risher et al., 2014; Bello-Medina et al., 2016). In addition, the rats for HE and Nissl staining were perfused with freshly prepared 4% paraformaldehyde in PBS immediately after PBS perfusion. Then, the brains were embedded in OCT compound (Tissue-Tek, Miles) at −20°C. Later, the PFC was sectioned into 15-μm thick slices in coronal plane with by Cryostat Microtome (Leica CM1860UV, Germany).

### Western Blotting Analysis

After the above test, the other rats were also sacrificed and perfused with PBS, and their mPFC was removed at 0°C and stored at −80°C. The procedure of western blotting analysis was described as previously published (Wang et al., 2018). The primary antibodies used in this study included the following: (1) inflammatory protein: anti-TLR4 (1:1000, Wanleibio, WL00196), anti-JNK (1:1000, Wanleibio, WL01295), anti-p-JNK (1:1000, CST, 4668), anti-NF-κB (1:1000, Abcam, ab16502), anti-IL-6 (1:1000, Wanleibio, WL02841); (2) synaptic functional protein: anti-SYP (1:2000, Abcam, ab32594), anti-NR2B (1:1000, Abcam, ab65783) and PSD95 (1:2000, GeneTex, GTX133091), anti-CaMKII (1:1000, Abcam, ab134041); (3) anti-GAPDH (1:1000, GeneTex, GTX627408). The secondary antibodies used in this study included (1) rabbit IgG antibody (HRP) (1:5000, GeneTex, GTX213110-01); (2) mouse IgG antibody (HRP) (1:5000, GeneTex, GTX213111-01).

### Statistical Analysis

All data were analyzed by IBM SPSS Statistics 20 software. The results of rats’ weight and delayed nonmatch-to-sample task were analyzed using repeated measures ANOVA. The other results were all analyzed by one-way ANOVA. LSD multiple-comparison test was performed for comparisons between groups. Data are expressed as the average ± standard error of the mean (SEM). The value of *p* < 0.05 is considered to be significant.

## Results

### Low-Intensity Focused Ultrasound Stimulation Ameliorates Working Memory Dysfunctions Induced by Vascular Dementia

**FIGURE 2:**
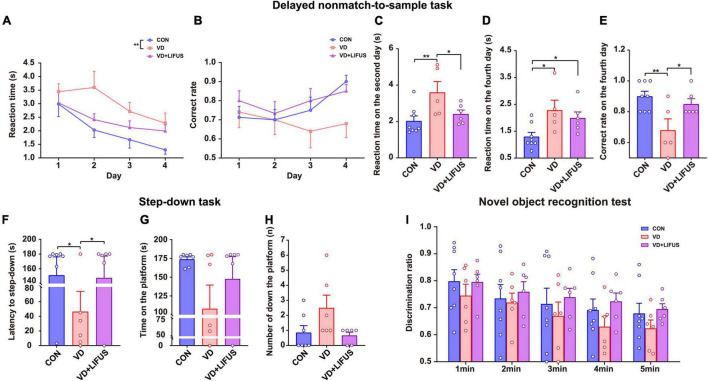
Effect of LIFUS on working memory dysfunctions in the VD rats. **(A–E)** The average response time of 4-day task **(A)**, average accuracy of 4-day task **(B)**, average reaction time on day 2 **(C)**, average reaction time on day 4 **(D)**, and average correct rate on day 4 **(E)** in the delayed nonmatch-to-sample task (CON, *n* = 8; VD, *n* = 5; VD + LIFUS, *n* = 6). **(F–H)** The latency of the step-down **(F)**, time on the platform **(G)**, and number of down the platform **(H)** in the step-down task (CON, *n* = 8; VD, *n* = 5; VD + LIFUS, *n* = 6). **(I)** The discrimination ratio for new objects during the test period 1–5 min in the novel object recognition test. Data are expressed as mean ± SEM. *p < 0.05, **p < 0.01 (CON, *n* = 8; VD, *n* = 6; VD + LIFUS, *n* = 6).

To verify whether LIFUS could cause adverse reactions in rats, we tested the weight changes in the rats during the experiment and detected the anxiety-like behavior of rats through the elevated-plus maze task. The result of weight changes showed that during the whole experiment, the weight of the three groups showed a similar increasing trend, and there was no statistical difference among the three groups (Figure 1D). At the same time, the results of elevated-plus maze task showed no significant difference among the three groups, which indicates that VD modeling and LIFUS process did not induce anxiety behavior in rats (Figures 1E,F). In addition, we tested the performance of rats in different working memory tasks to verify the effect of LIFUS on working memory impairments in VD rats. The results of different working memory tasks showed that LIFUS significantly improved the performance of working memory tasks in VD rats, which includes the delayed nonmatch-to-sample task and step-down task (Figure 2). In the delayed nonmatch-to-sample task, the response time gradually decreased with the increase of training times, and there was significant difference among the three groups (repeated measures ANOVA, *F*(2, 16) = 5.196, *p* = 0.018). Compared with CON group, the response time of VD group was significantly longer (*p* = 0.005) (Figure 2A). On the 2nd day, the response time of VD group was longer than that in the other groups (one-way ANOVA, *F*(2,16) = 4.908, *p* = 0.022; CON vs. VD: *p* < 0.01; VD vs. VD + LIFUS: *p* < 0.05) (Figure 2C). On the 4th day, the response time of both VD and VD + LIFUS group was longer than that in the CON group (*p* < 0.05) (Figure 2D). Moreover, the increasing trend of correct rate with the increase of training times was similar among the three groups, and no significant difference was found (Figure 2B). However, on the 4th day, the VD group had a significantly poor performance on making the right choice than other groups (one-way ANOVA, *F*(2,16) = 6.063, *p* = 0.011; CON vs. VD: *p* < 0.01; VD vs. VD + LIFUS: *p* < 0.05) (Figure 2E). The results of step-down task also showed the LIFUS improved the working memory impairment induced by VD. The VD group had a poor performance in step-down task that the latency of VD group was significantly shorter than CON group, whereas performance of VD + LIFUS group was reversed (one-way ANOVA,*F*(2,16) = 4.672,*p* = 0.025; CON vs. VD: *p* < 0.05; VD vs. VD + LIFUS: *p* < 0.05) (Figure 2F). There were no significant differences in other indicators of step-down task (Figures 2G,H), nor in the novel object recognition test (Figure 2I). Taken together, these results of behavioral tests suggest that LIFUS can significantly ameliorate working memory dysfunctions induced by VD without inducing adverse reactions such as weight loss and anxiety-like behavior.

### Low-Intensity Focused Ultrasound Stimulation Increases Cerebral Blood Flow in the Vascular Dementia Rats

**FIGURE 3:**
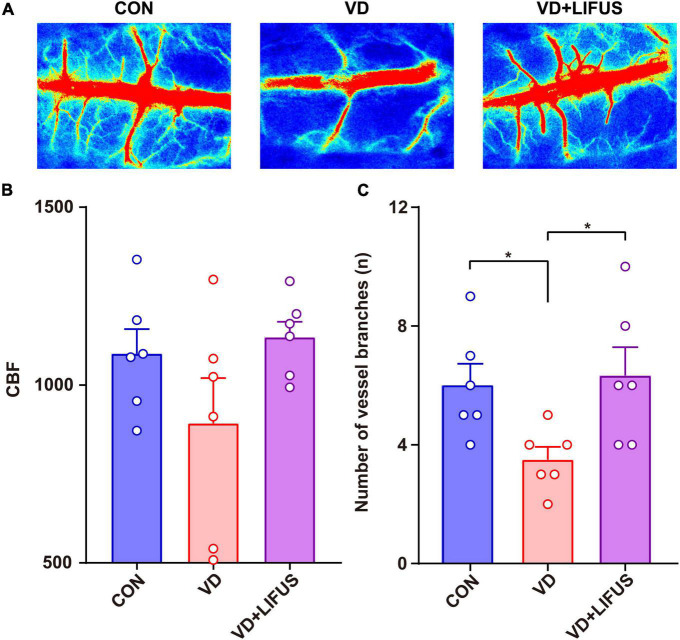
Effect of LIFUS on blood flow in VD rats. **(A)** Typical diagrams of brain flow in the region of interest in three groups. **(B)** The average blood flow of main vein. **(C)** The number of vascular branches. Data are expressed as mean ± SEM. *p < 0.05 (CON, VD, VD + LIFUS, *n* = 6).

The CBF was measured to test the effect of LIFUS on global cerebral ischemia in VD rats. The results are shown in the Figure 3. Figure 3A shows the typical CBF imaging of three groups. There were abundant vascular branches in CON and VD + LIFUS group, not VD group. The statistical results of the blood flow of the main vein and number of vascular branches are shown in the Figures 3B,C, respectively. Although there was difference in blood flow of the main vein among three groups, they were not significant (Figure 3B). Compared with CON and VD + LIFUS group, the number of vascular branches in VD groups decreased significantly (one-way ANOVA, F (2, 15) = 4.420, *p* = 0.031; CON vs. VD: *p* < 0.05; VD vs. VD + LIFUS: *p* < 0.05) (Figure 3C). These results indicate that LIFUS targeting the mPFC can increase global CBF in VD rats and suggest that the improvements of working memory in VD rats by the LIFUS may be associated with increased vascular branches and blood flow.

### Low-Intensity Focused Ultrasound Stimulation Improves the Survivability of the Neurons and Ability of Protein Synthesis in the Vascular Dementia Rats

**FIGURE 4:**
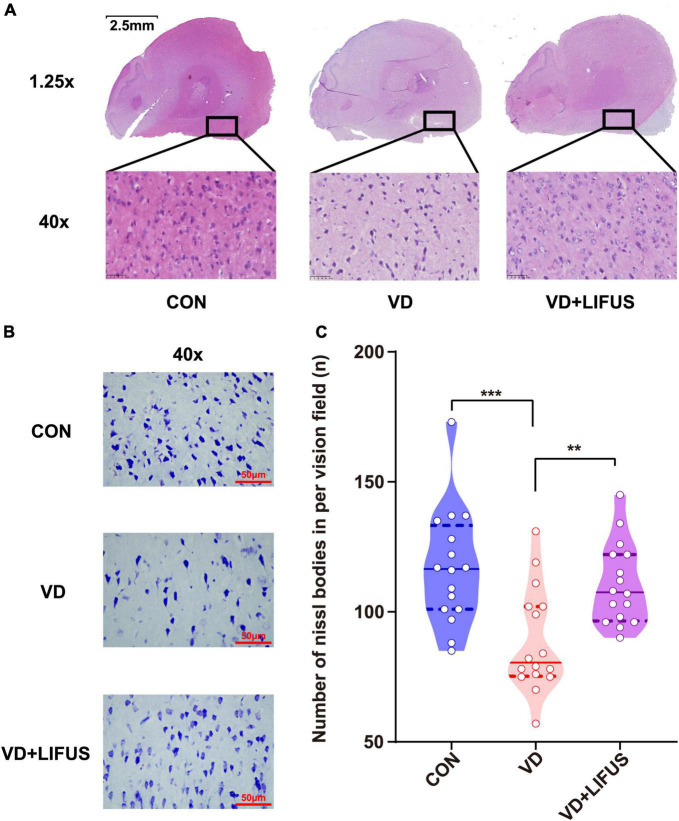
Effect of LIFUS on the morphological changes of the neurons and ability of protein synthesis in the VD rats. **(A)** Typical diagrams of HE in three groups. **(B)** Typical diagrams of Nissl staining in three groups. **(C)** The statistical results of the number of Nissl bodies. Data are expressed as mean ± SEM. **p < 0.01, ***p < 0.001 (CON, VD, VD + LIFUS, *n* = 2).

The HE was performed to test the effect of LIFUS on the morphological changes of neurons in the rats. The number of nucleus in the PFC of VD group significantly reduced, and the arrangement of neurons was loosen and pyknosis appeared compared with CON group, whereas these were all reversed after LIFUS (Figure 4A). In addition, to evaluate the effects on protein synthesis of neurons in the PFC after ultrasound treatment, we observed the Nissl body expression in different groups. The Figure 4B shows the typical expression of Nissl bodies in PFC of the three groups. We can observe that compared with the other groups, the distribution of Nissl bodies in VD group was looser. Additionally statistically, the number of Nissl bodies in VD group was significantly decreased (one-way ANOVA, *F*(2,45) = 9.318, *p* < 0.001; CON vs. VD: *p* < 0.001; VD vs. VD + LIFUS, *p* = 0.002) (Figure 4C). The above results show that the LIFUS can enhance the survival ability and protein synthesis ability of neurons in PFC of VD rats.

### Low-Intensity Focused Ultrasound Stimulation Enhances the Density of Dendritic Spines and Expression of Synaptic Proteins in the Vascular Dementia Rats

**FIGURE 5:**
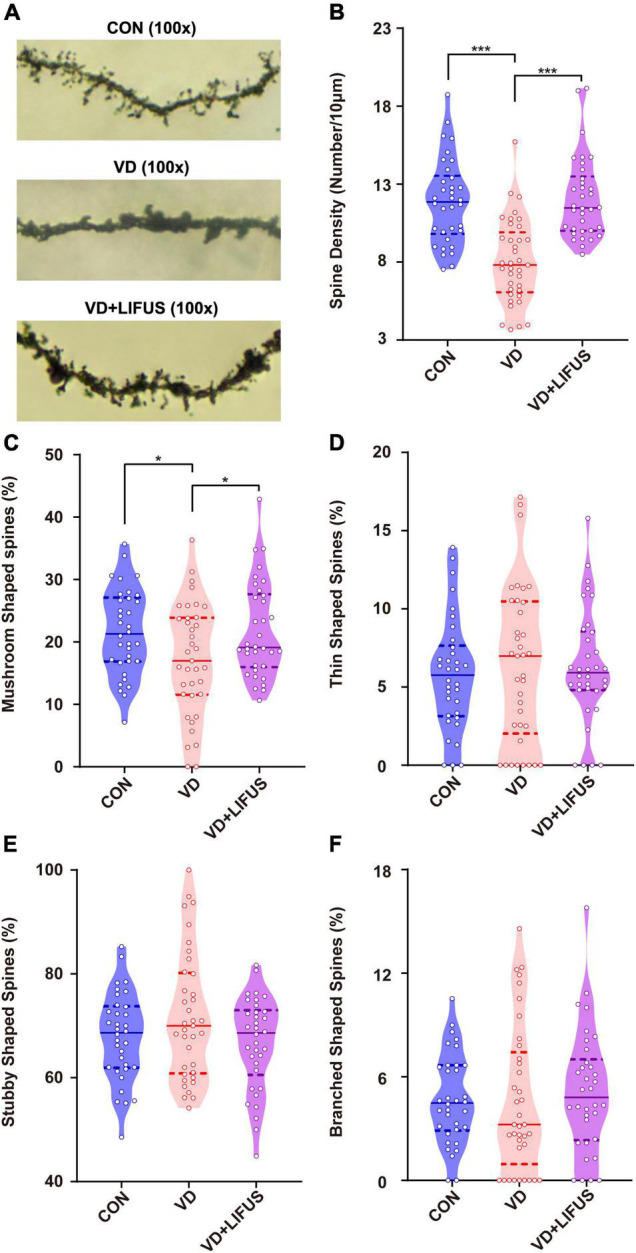
Effect of LIFUS on the density of dendritic spines. **(A)** Typical diagrams of Golgi-cox staining in three groups. **(B)** The spine density in three groups. **(C–F)** The percentage of the mushroom shaped spines, thin shaped spines, stubby shaped spines, and branched shaped spines. Data are expressed as mean ± SEM. *p < 0.05, *** *p* < 0.001 (CON, *n* = 6; VD, *n* = 4; VD + LIFUS, *n* = 4).

**FIGURE 6:**
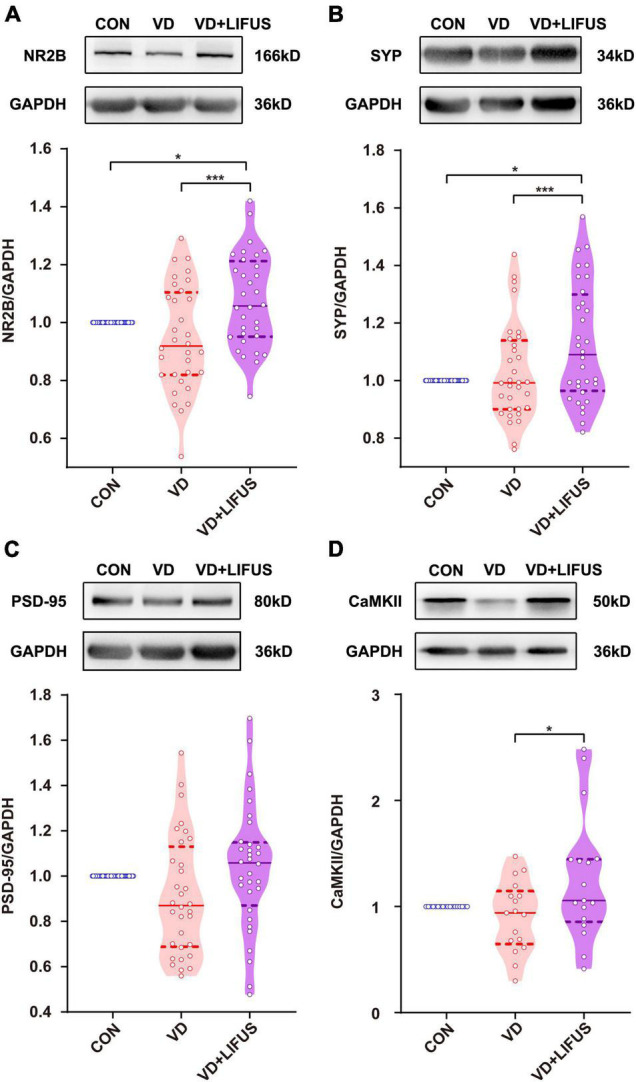
Effect of LIFUS on the expression of synaptic proteins. **(A–D)** The expression of NR2B, SYP, PSD-95, and CaMKII. Data are expressed as mean ± SEM. *p < 0.05, *** *p* < 0.001 (CON, *n* = 6; VD, *n* = 4; VD + LIFUS, *n* = 4).

Next, we examined the effects of LIFUS on synaptic structure and expression of synaptic proteins. The typical examples of expression of dendritic spines in the PFC of the three groups showed that the density of dendritic spines in VD group was less than that in CON and VD + LIFUS group (Figure 5A). This point was further proved by the statistical results (one-way ANOVA, *F*(2,102) = 20.311, *p* < 0.001; CON vs. VD, *p* < 0.001; VD vs. VD + LIFUS, *p* < 0.001) (Figure 5B). By analyzing the proportion of various subtypes of dendritic spines, we found that the proportion of mushroom type, not other subtypes, in VD group was significantly lower than other two groups (one-way ANOVA, *F*(2,102) = 3.907, *p* = 0.023; CON vs. VD, *p* = 0.022; VD vs. VD + LIFUS, *p* = 0.015) (Figures 5C–F). The Figures 6A–D show the statistical results of synaptic protein expression levels. Compared with CON group, the expression of NR2B, PSD-95, and CaMKII decreased in VD group without statistical difference. Moreover, the expression of NR2B, PSD-95, CaMKII, and SYP was increased in VD + LIFUS group, and there was significant difference between NR2B and SYP (NR2B: *F*(2,101) = 7.521, *p* = 0.001, CON vs. VD + LIFUS, *p* < 0.05; SYP: *F*(2,101) = 7.194, *p* = 0.001, CON vs. VD + LIFUS, *p* < 0.001). In addition, the expression of NR2B, CaMKII, and SYP in VD + LIFUS group was increased significantly than those in VD group (NR2B: *F*(2,101) = 7.521, *p* = 0.001, VD vs. VD + LIFUS, *p* < 0.001; CaMKII: *F*(2,101) = 3.738, *p* = 0.031, VD vs. VD + LIFUS, *p* < 0.05; SYP: *F*(2,101) = 7.194, *p* = 0.001, VD vs. VD + LIFUS, *p* = 0.007). The above results show that the LIFUS can increase the density of dendritic spines and the proportion of mushroom shaped dendritic spines and improve the expression of synaptic proteins in the mPFC of VD rats.

### Low-Intensity Focused Ultrasound Stimulation Inhibits the TLR4/NF-κB Pathway and Decreases Proinflammatory Cytokines in the Vascular Dementia Rats

**FIGURE 7:**
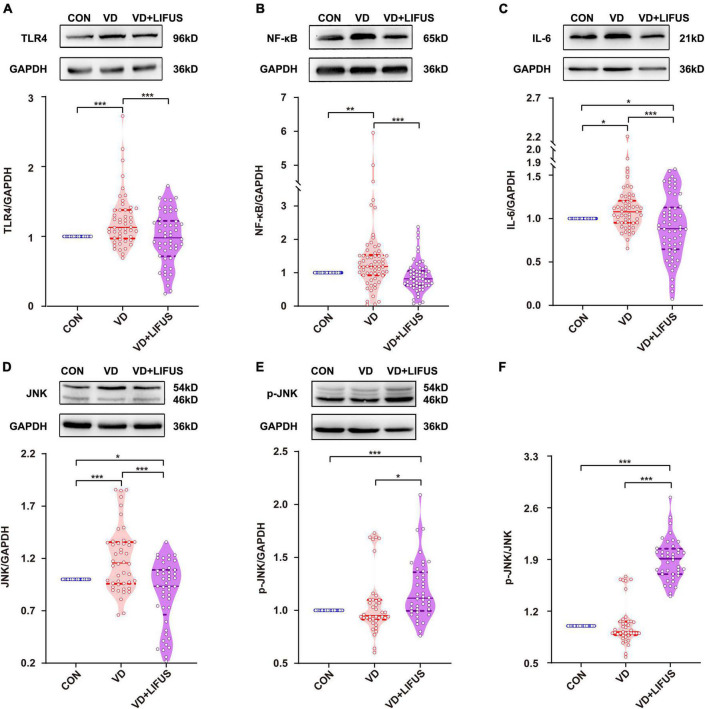
Effect of LIFUS on the expression of neuroinflammatory related proteins. **(A–F)** The expression of TLR4, NF-κB, IL-6, JNK, p-JNK, and ratio of p-JNK/JNK. Data are expressed as mean ± SEM. *p < 0.05, **p < 0.01, ***p < 0.001 (CON, *n* = 6; VD, *n* = 4; VD + LIFUS, *n* = 4).

After that, we detected whether the TLR4/NF-κB pathway and proinflammatory cytokines were involved the improvements of working memory in VD rats by the LIFUS. As shown in Figures 7A–F, the protein expression levels of TLR4, JNK, NF-κB, and the proinflammatory IL-6 in mPFC of VD group were significantly increased compared with these in CON group (TLR4: *F*(2,151) = 10.404, *p* < 0.001, CON vs. VD, *p* < 0.001; JNK: *F*(2,136) = 19.521, *p* < 0.001, CON vs. VD, *p* < 0.001; NF-κB: *F*(2,178) = 10.167, *p* < 0.001, CON vs. VD, *p* < 0.001; IL-6: *F*(2,169) = 9.130, p < 0.001, CON vs. VD, *p* < 0.05). Meanwhile, the LIFUS significantly inhibited the expression of these proteins in mPFC (TLR4: VD vs. VD + LIFUS, *p* < 0.001; JNK: VD vs. VD + LIFUS, *p* < 0.001; NF-κB: VD vs. VD + LIFUS, *p* < 0.001; IL-6: VD vs. VD + LIFUS, *p* < 0.001). However, the p-JNK and ratio of p-JNK/JNK in VD + LIFUS group were significantly higher than the other group (p-JNK: *F*(2,117) = 10.404, *p* < 0.001, CON vs. VD + LIFUS, *p* < 0.001, VD vs. VD + LIFUS, *p* < 0.05; p-JNK/JNK: *F*(2,117) = 19.521, *p* < 0.001, CON vs. VD + LIFUS, *p* < 0.001, VD vs. VD + LIFUS, *p* < 0.001) (Figures 7A–F). The above results show that the LIFUS can decrease proinflammatory cytokines, such as IL-6, by inhibiting the TLR4/NF-κB pathway in the VD rats.

## Discussion

In this study, we first provides the experimental evidence that LIFUS, targeting the mPFC, markedly improves specific working memory impairments in VD rats. Furthermore, our results support the hypotheses that promoting CBF and antiinflammatory may be the potential mechanisms for ameliorating the cognitive impairments induced by VD.

There is extensive research on ultrasound stimulation treating the cognitive impairments in different animal models (Huang et al., 2017; Eguchi et al., 2018; Chen et al., 2019). These researches suggested that LIFUS could affect the different cognitive functions while stimulating different brain regions involved in different cognitive processes. Therefore, the targeted brain region is one of the key factors that affect the efficacy of LIFUS. It has been recognized that mPFC is essential for social, affective functions and cognitive process including working memory (Etkin et al., 2011; Euston et al., 2012; Hiser and Koenigs, 2018; Xu et al., 2019). Meanwhile, excitation of the PFC using brain stimulation technology, such as transcranial current stimulation, optogenetic and chemogenetic neuromodulation, can regulate the working memory *via* modulating the neural activities and neural oscillations in the PFC (Alekseichuk et al., 2016; Abbas et al., 2018; Nagai et al., 2020; Upright and Baxter, 2020). Thus, mPFC might be an effective targeted brain region, which we focused in this study to investigate the neural modulation function of LIFUS. However, it was still not clear the specific action of LIFUS under our experimental condition. Therefore, three kinds of behavioral tasks were carried out to evaluate the different types working memory. Interestingly, we found that LIFUS could affect the reward-based spatial working memory and fear working memory task but not object recognition memory. We speculated that it might be associated with the decision-making on risk and reward function of mPFC (Der-Avakian and Markou, 2012; Euston et al., 2012; Pitts et al., 2016; Hiser and Koenigs, 2018; Murray and Rudebeck, 2018) and negative emotion including fear as well (Etkin et al., 2011; Euston et al., 2012; Giustino and Maren, 2015; Hiser and Koenigs, 2018). These functions of mPFC are believed to rely on the neural activities and neural oscillations during distinct task processes (Der-Avakian and Markou, 2012; Eriksson et al., 2015; Giustino and Maren, 2015; Riley and Constantinidis, 2015; Murray and Rudebeck, 2018). Additionally, a series of studies found that ultrasound stimulation, including focused ultrasound and transcranial pulsed ultrasound stimulation, could stimulate intact brain circuits and modulate the neuronal oscillations (Tufail et al., 2010; Yuan et al., 2016a,b). So, we believe that the improvements in specific working memory impairments may be due to the regulation of neural oscillations in mPFC by LIFUS, although this needs to be verified in the subsequent experiments.

As discussed above, changes in neural activities and neural oscillations could affect working memory. The origin of them thus become important as well which depend on synaptic function. So, we evaluated the changes of synaptic function by analyzing the synaptic structure and synaptic protein (Wang et al., 2013). In previous study, it has been reported that the LIFUS could modulate structural and functional synaptic plasticity in rat hippocampus by increasing the density of dendritic spines and the expression level of GluN2A (Huang et al., 2019). Consistent with previous results, we found that the LIFUS not only improved the density of dendritic spines and the expression of synaptic proteins, including the NR2B, PSD-95, CaMKII, and SYP, but also improved the ability of protein synthesis in the mPFC of VD rats. Hence, we believed that the improvements in synaptic structure and synaptic protein contributed to facilitate neural activity in mPFC, thereby improving cognitive behavior.

Then, how LIFUS could improve synaptic function? Previous studies have demonstrated that angiogenesis and antiinflammation may be the two neuroprotection effects activated by the ultrasound stimulation (Kim et al., 2017; Li et al., 2017; Liu et al., 2017; Eguchi et al., 2018; Chen et al., 2019; Chang et al., 2020). Several experiments suggested that LIFUS could lead to angiogenesis in VD and cerebral ischemia rodents, thereby promoting the survival of neurons (Li et al., 2017; Eguchi et al., 2018). On the other hand, a serious of studies found that ultrasound stimulation suppresses LPS-induced proinflammatory responses by regulating TLR4/NF-κB pathway and release of proinflammatory cytokines *in vivo* and *in vitro* (Liu et al., 2017; Chen et al., 2019; Chang et al., 2020). Therefore, we explored the underlying mechanism from CBF and neuroinflammation, respectively. Our results about CBF and neuroinflammation were in agreement with those of previous studies. On the one hand, we found that LIFUS increased vascular branches and blood flow in mPFC, which is consistent with previous results. Hence, the alleviated hemodynamic compromise and the improved CBF observed in this study should be one mechanism of LIFUS treating the VD. On the other hand, the past findings are consistent with our results that the expressions of TLR4, NF-κB, and IL-6 decreased significantly after LIFUS treatment. Hence, antiinflammation may be another neuroprotection mechanism of LIFUS treating the VD. However, there is a difference that p-JNK expression was significantly increased after LIFUS treatment. This disparity may be due to the reason that JNK, the activities of which are stimulated by many physiological, pathological, and environmental cues, are involved in diverse and sometimes even opposing cell functions (Xia and Karin, 2004). The previous studies have found that the significant upregulation of p-JNK2 could enhance the proliferation and neuronal differentiation of rat embryonic neural stem cells (Jiao et al., 2017). Additionally, the ischemia –hypoxia could activate JNK signaling and results in proliferation of neural stem cells (Chen et al., 2010; Cai et al., 2014). Meanwhile, ultrasound stimulation has been found to promote the proliferation of HaCaT keratinocytes, osteoblasts, and human chondrocyte cell by activating JNK (Choi et al., 2007; Chiu et al., 2008; Leng et al., 2018). LIFUS used in this study may be induced the proliferation of neural cells *via* activating JNK, although the exact mechanisms and pathways remain to be investigated.

**FIGURE 8:**
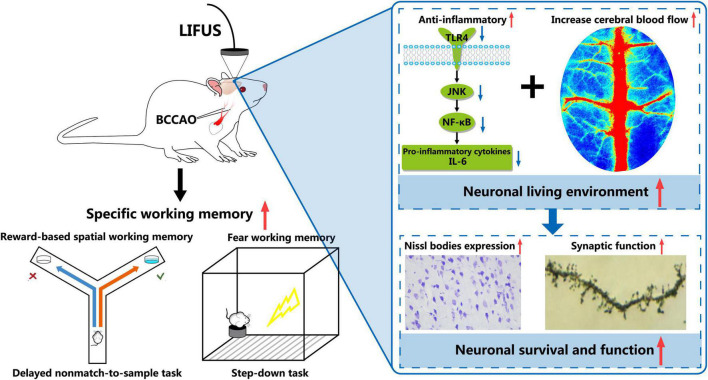
Schematic diagram of the potential mechanism involved in LIFUS treating the working memory disorders induced by VD.

Overall, as shown in Figure 8, we demonstrated that LIFUS treatment significantly ameliorated the reward and fear-based working memory dysfunctions, the survivability of the neurons, and synaptic functions in VD rats. These beneficial effects of LIFUS might be due to the increase in the CBF and inhibit the TLR4/NF-κB pathway. Our findings indicated that LIFUS could be a novel therapeutic technique for the treatment of central nervous system diseases related to cognitive impairments.

## Data Availability Statement

The original contributions presented in the study are included in the article/Supplementary Material, further inquiries can be directed to the corresponding authors.

## Ethics Statement

The animal study was reviewed and approved by Animal Research Ethics Committee of Tianjin Hospital.

## Author Contributions

FW performed the surgery, behavioral test, LIFUS procedure, and wrote the manuscript. QW performed the all experiments and analyzed the data. LW and JR contributed to behavioral test and the western blotting experiment. XS contributed to the LIFUS procedure. YT and CZ contributed to design the study and revised the manuscript. CZ, YT, and JY obtained the funding. JY and DM designed the study, revised the manuscript, and gave final approval of the manuscript. All authors contributed to the article and approved the submitted version.

## Conflict of Interest

The authors declare that the research was conducted in the absence of any commercial or financial relationships that could be construed as a potential conflict of interest.

## Publisher’s Note

All claims expressed in this article are solely those of the authors and do not necessarily represent those of their affiliated organizations, or those of the publisher, the editors and the reviewers. Any product that may be evaluated in this article, or claim that may be made by its manufacturer, is not guaranteed or endorsed by the publisher.
