# Supplementary figures and images for: Low-Intensity Focused Ultrasound Stimulation Ameliorates Working Memory Dysfunctions in Vascular Dementia Rats via Improving Neuronal Environment
Source: Front Aging Neurosci. 2022 Feb 21;14:814560. doi: 10.3389/fnagi.2022.814560 (PMC8899543; doi:10.3389/fnagi.2022.814560)

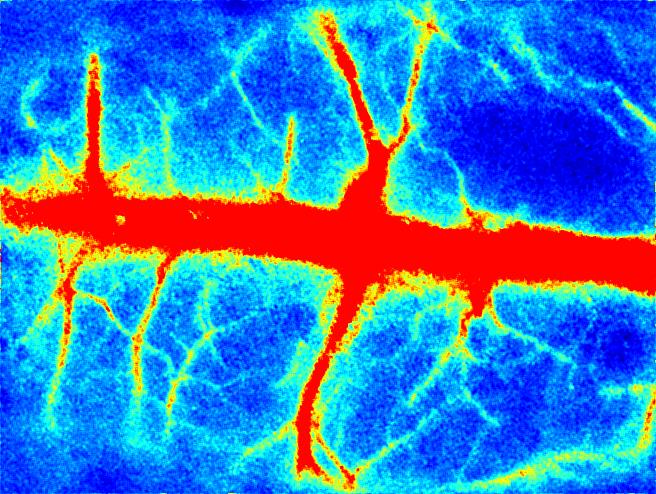

Supplement: Supplementary file 3 [file Data_Sheet_2.ZIP › Original images displayed inmanuscript-Cerebral Blood Flow/CON rat.jpg]

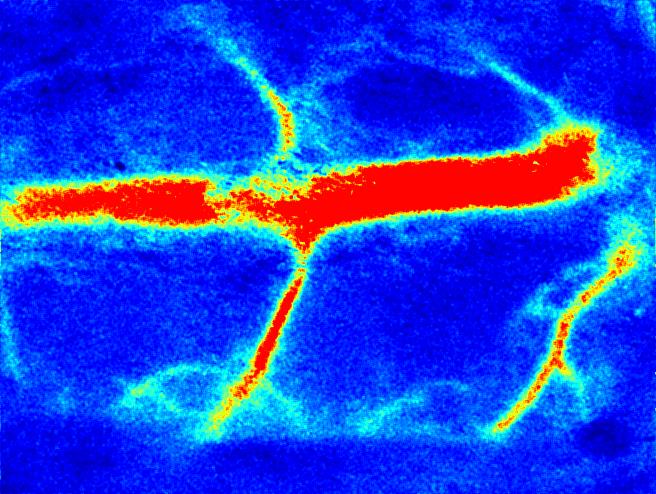

Supplement: Supplementary file 3 [file Data_Sheet_2.ZIP › Original images displayed inmanuscript-Cerebral Blood Flow/VD rat.jpg]

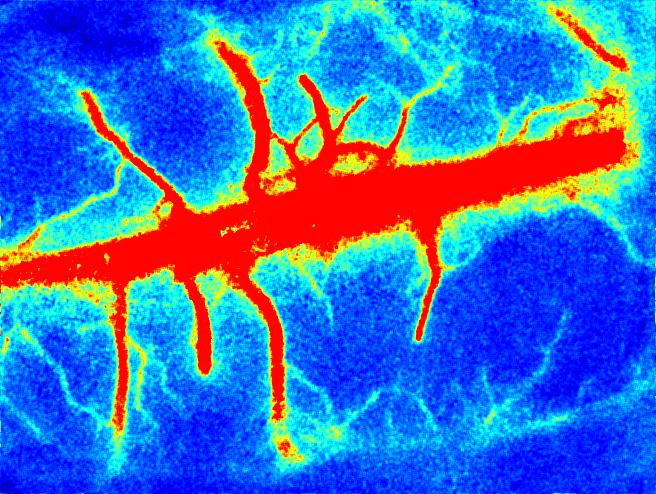

Supplement: Supplementary file 3 [file Data_Sheet_2.ZIP › Original images displayed inmanuscript-Cerebral Blood Flow/VD+LIFUS rat.jpg]

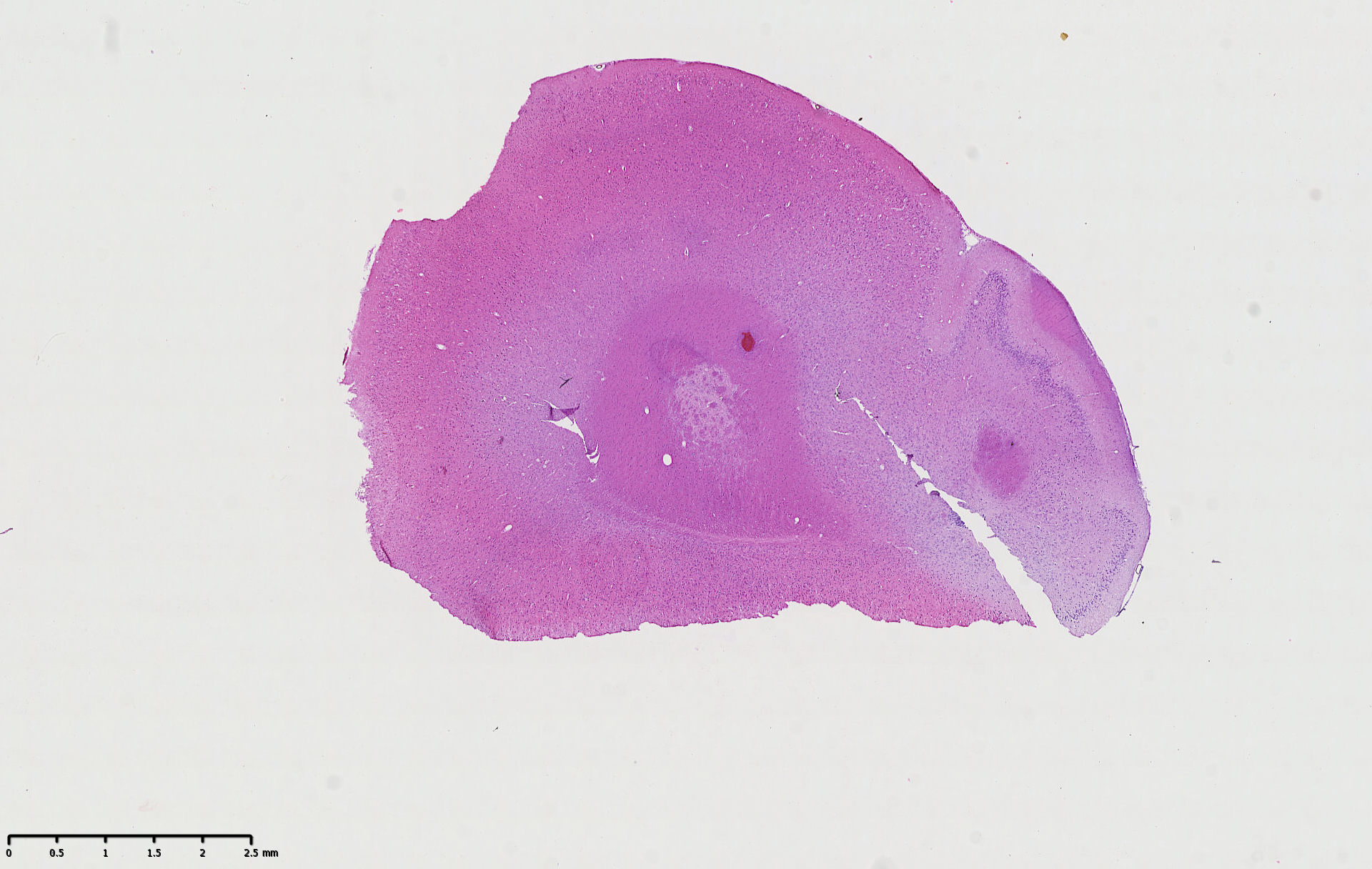

Supplement: Supplementary file 4 [file Data_Sheet_3.ZIP › Original images displayed inmanuscript-HE/CON rat-1.25x.tif]

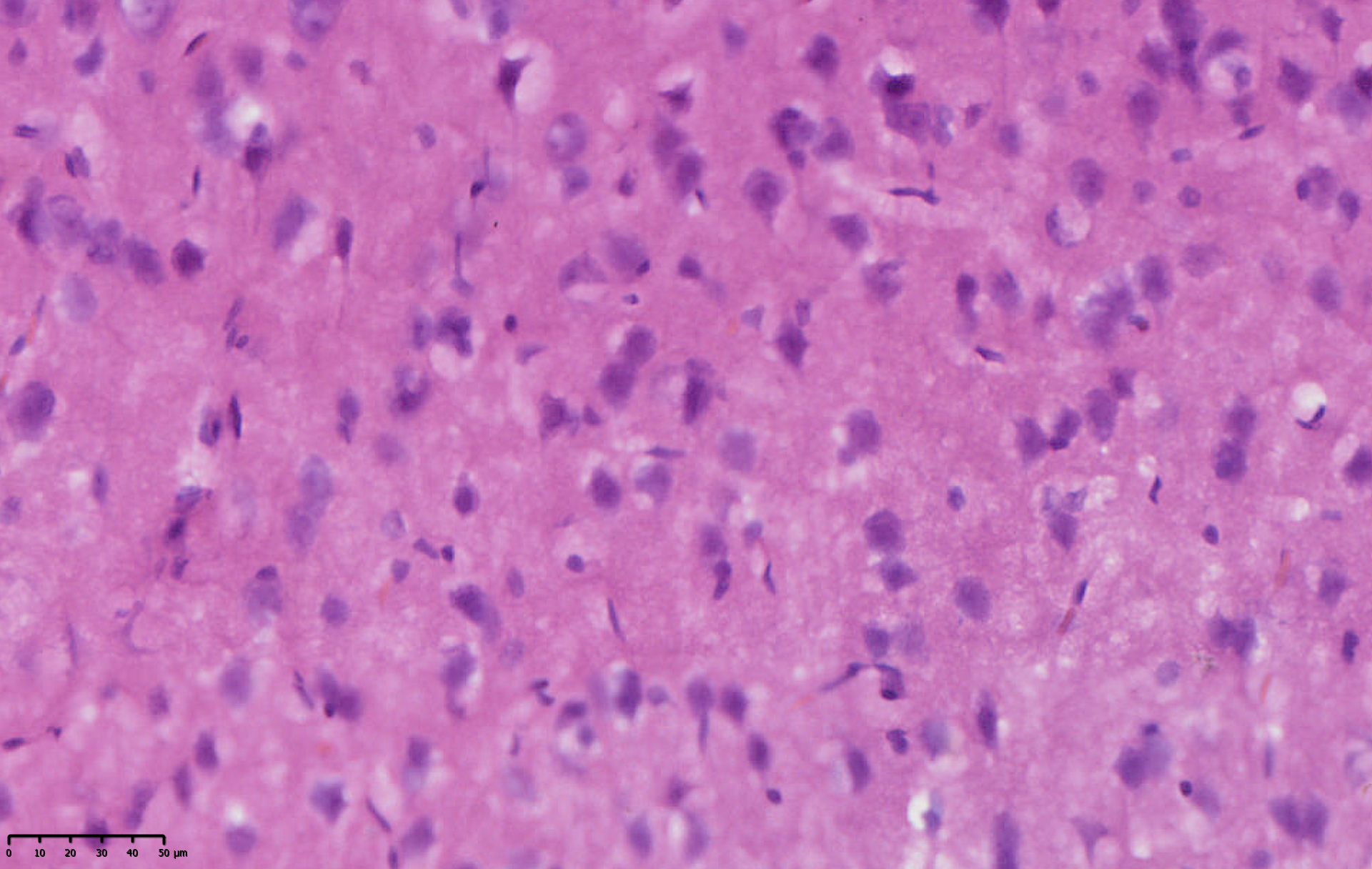

Supplement: Supplementary file 4 [file Data_Sheet_3.ZIP › Original images displayed inmanuscript-HE/CON rat-40x.tif]

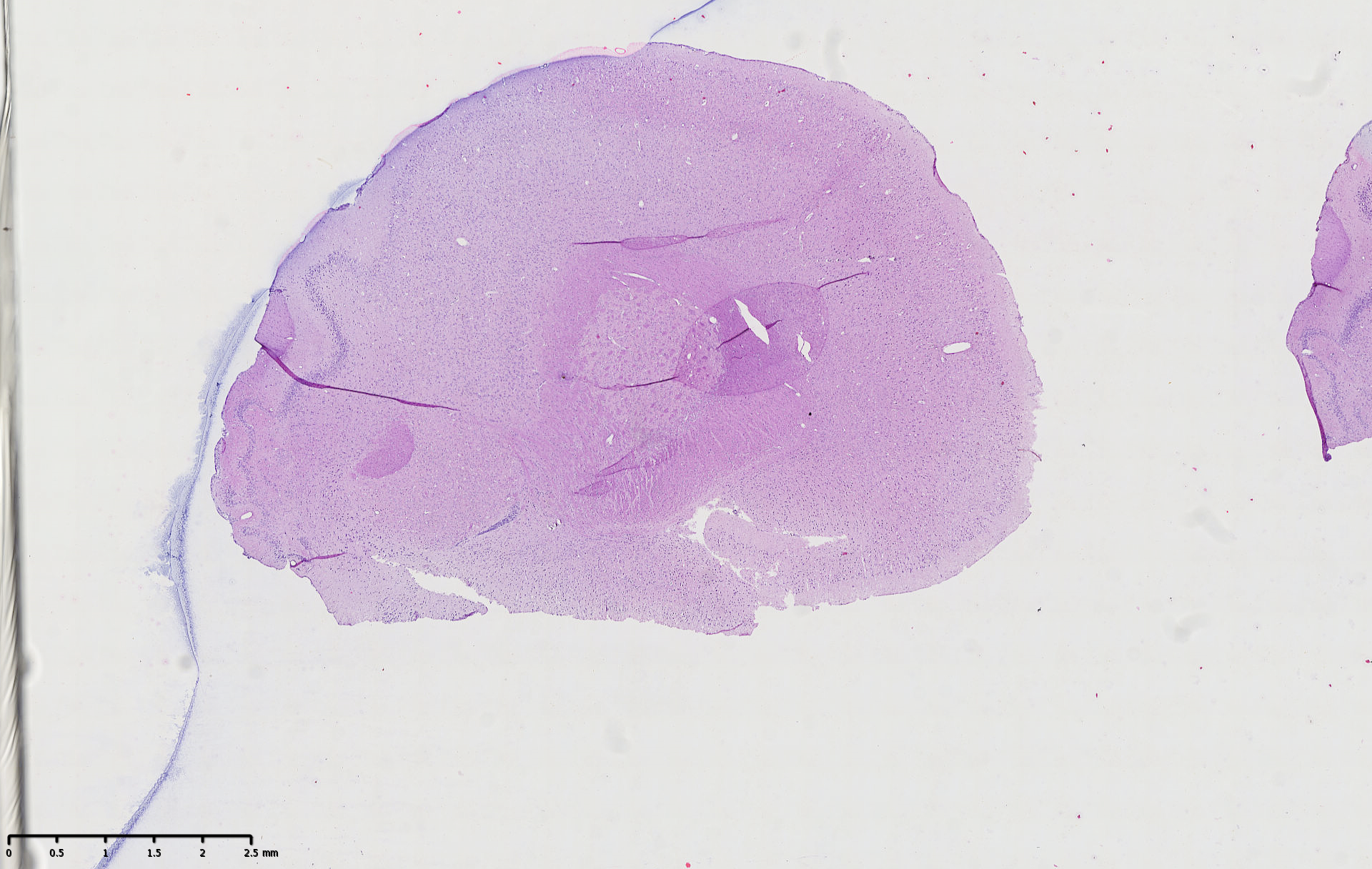

Supplement: Supplementary file 4 [file Data_Sheet_3.ZIP › Original images displayed inmanuscript-HE/VD rat-1.25x.tif]

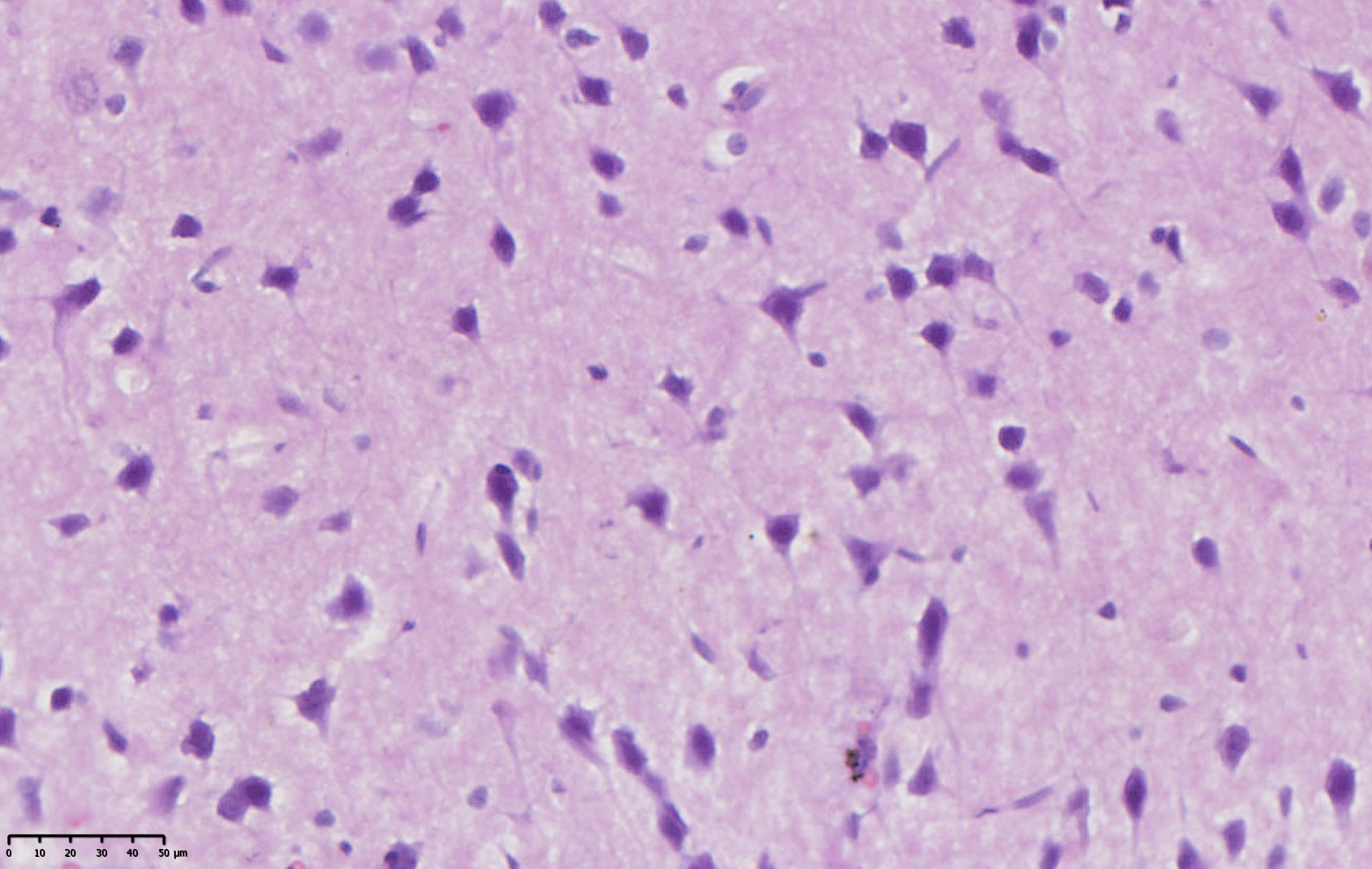

Supplement: Supplementary file 4 [file Data_Sheet_3.ZIP › Original images displayed inmanuscript-HE/VD rat-40x.tif]

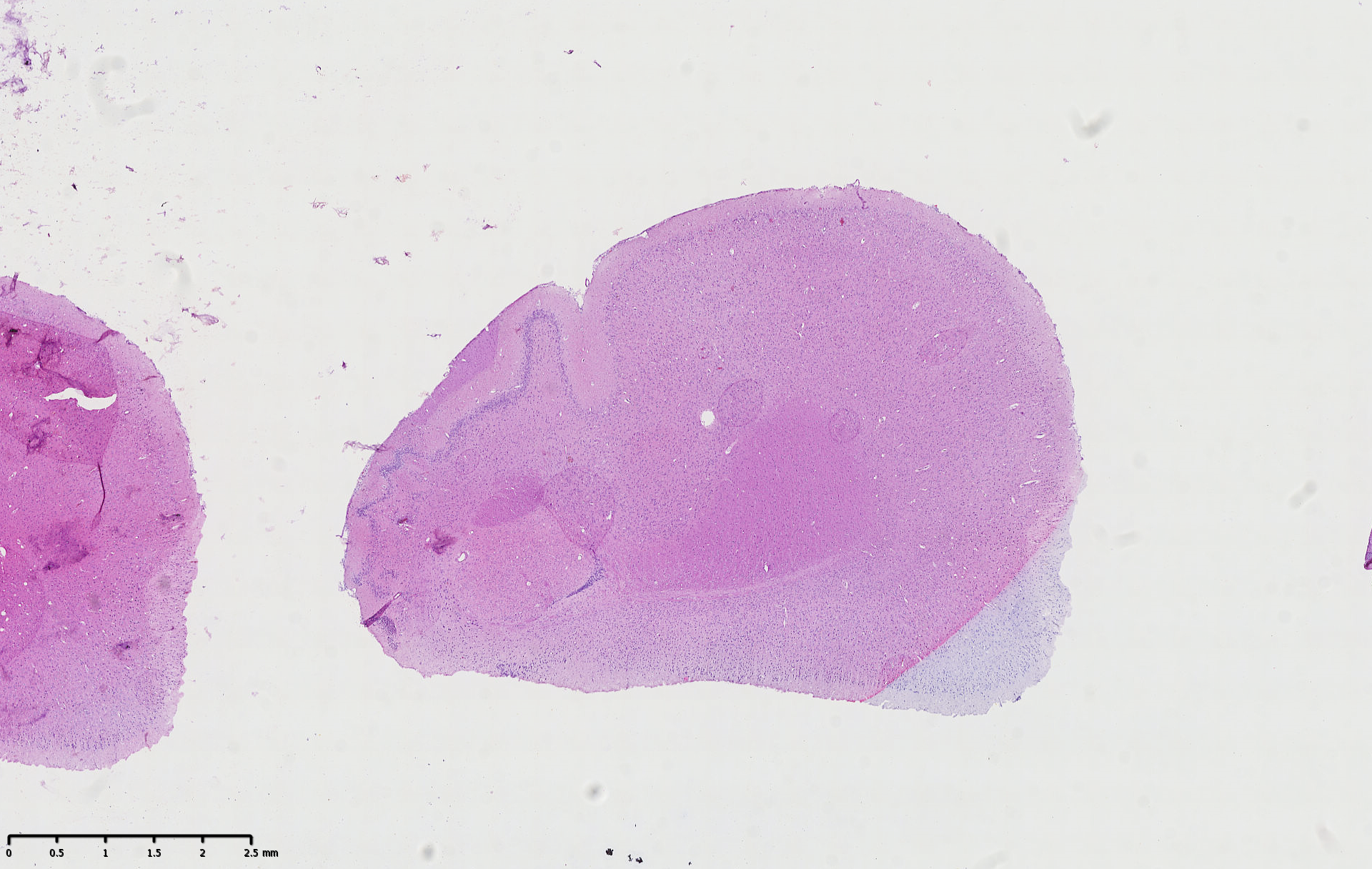

Supplement: Supplementary file 4 [file Data_Sheet_3.ZIP › Original images displayed inmanuscript-HE/VD+LIFUS rat-1.25x.tif]

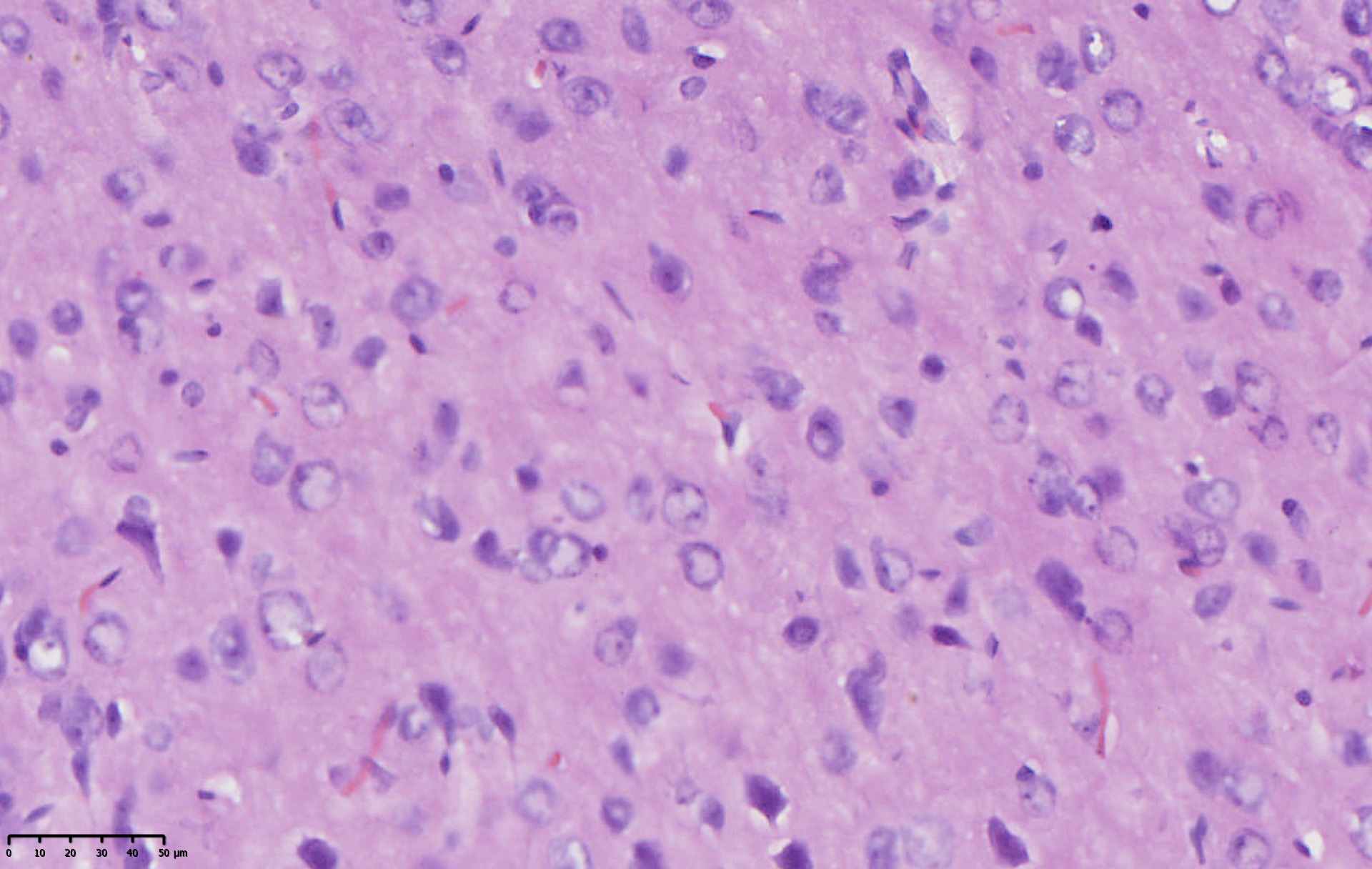

Supplement: Supplementary file 4 [file Data_Sheet_3.ZIP › Original images displayed inmanuscript-HE/VD+LIFUS rat-40x.tif]

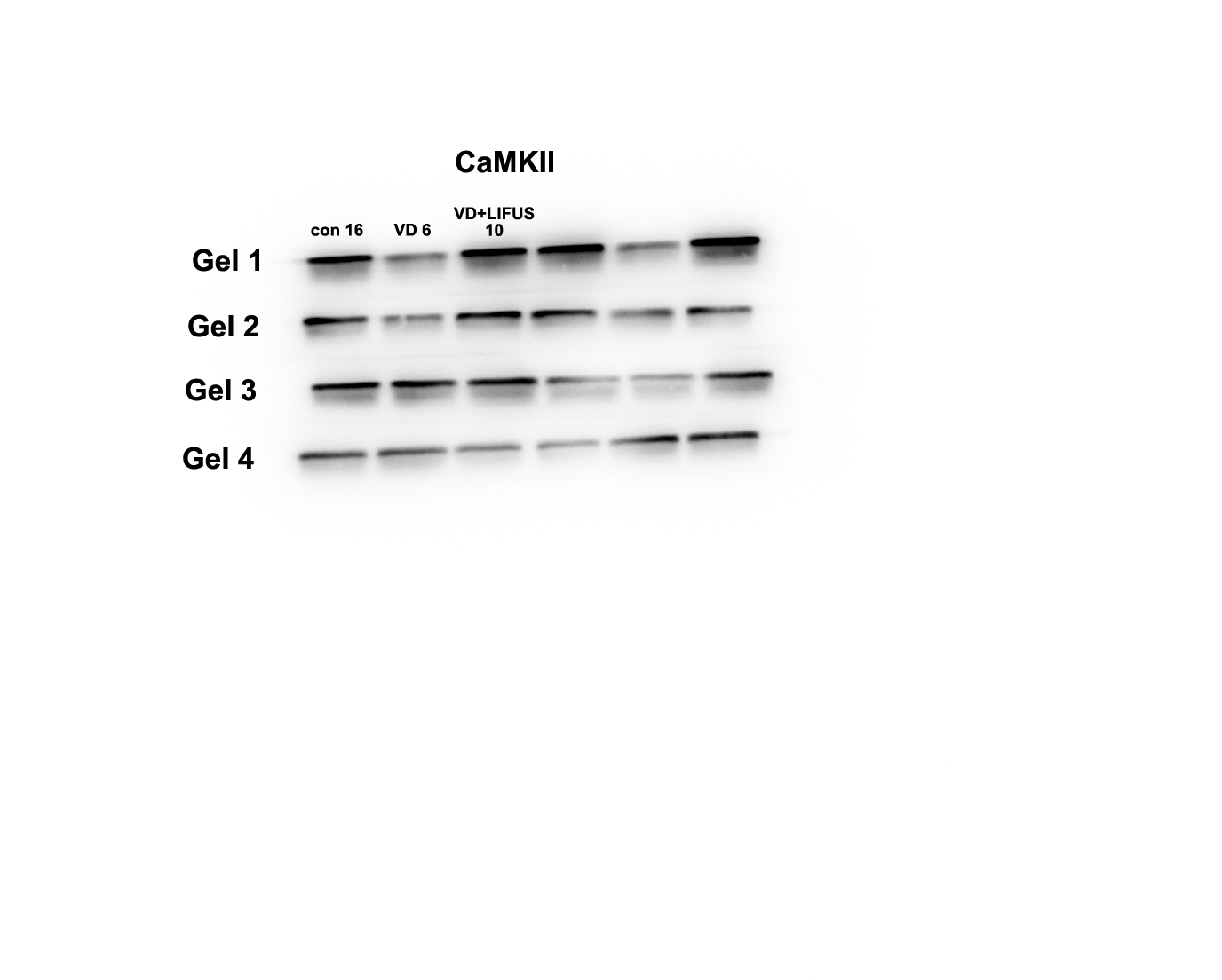

Supplement: Supplementary file 5 [file Data_Sheet_4.ZIP › Original images displayed inmanuscript-Western blot/CaMKII/CAMKII.tif]

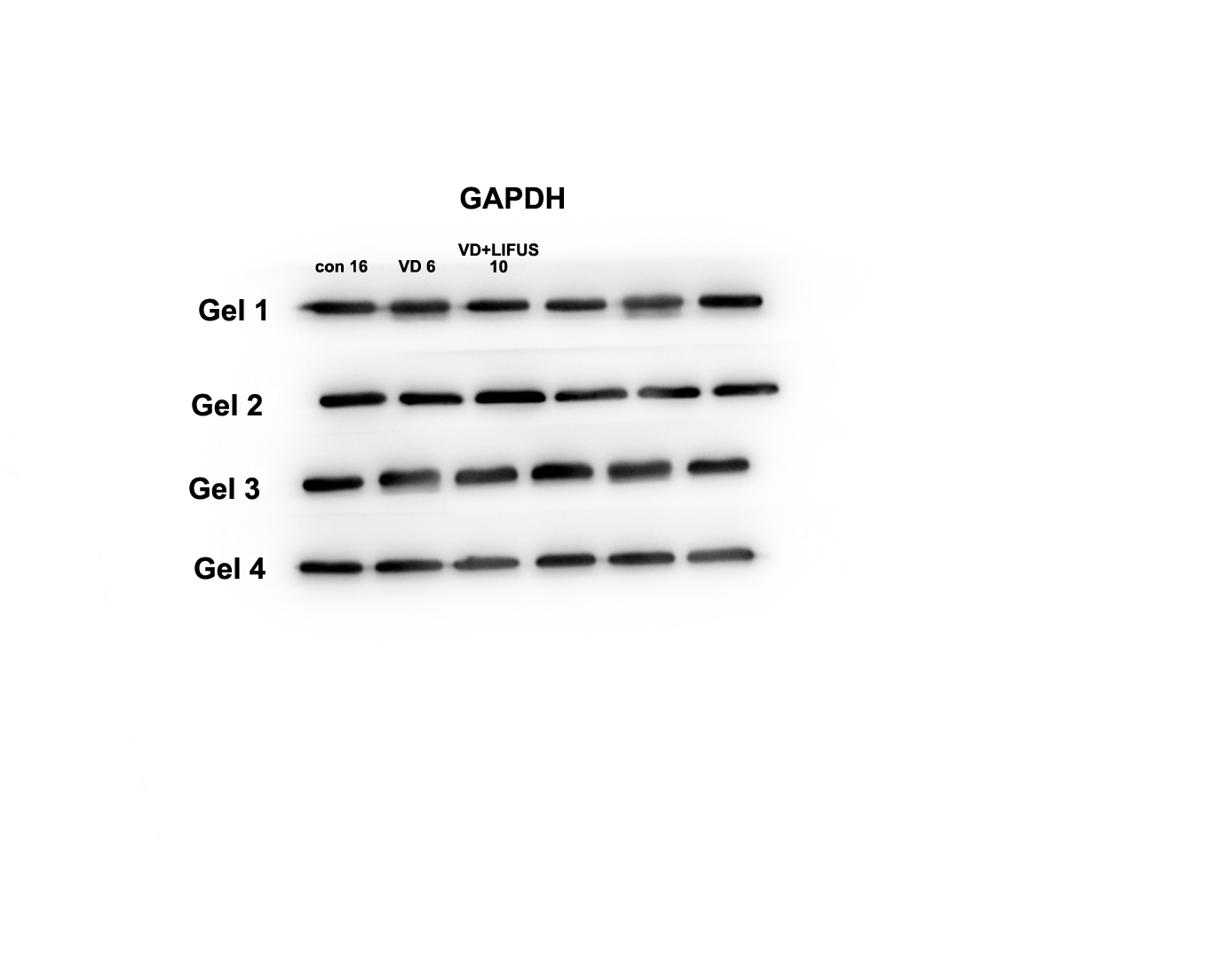

Supplement: Supplementary file 5 [file Data_Sheet_4.ZIP › Original images displayed inmanuscript-Western blot/CaMKII/GAPDH.tif]

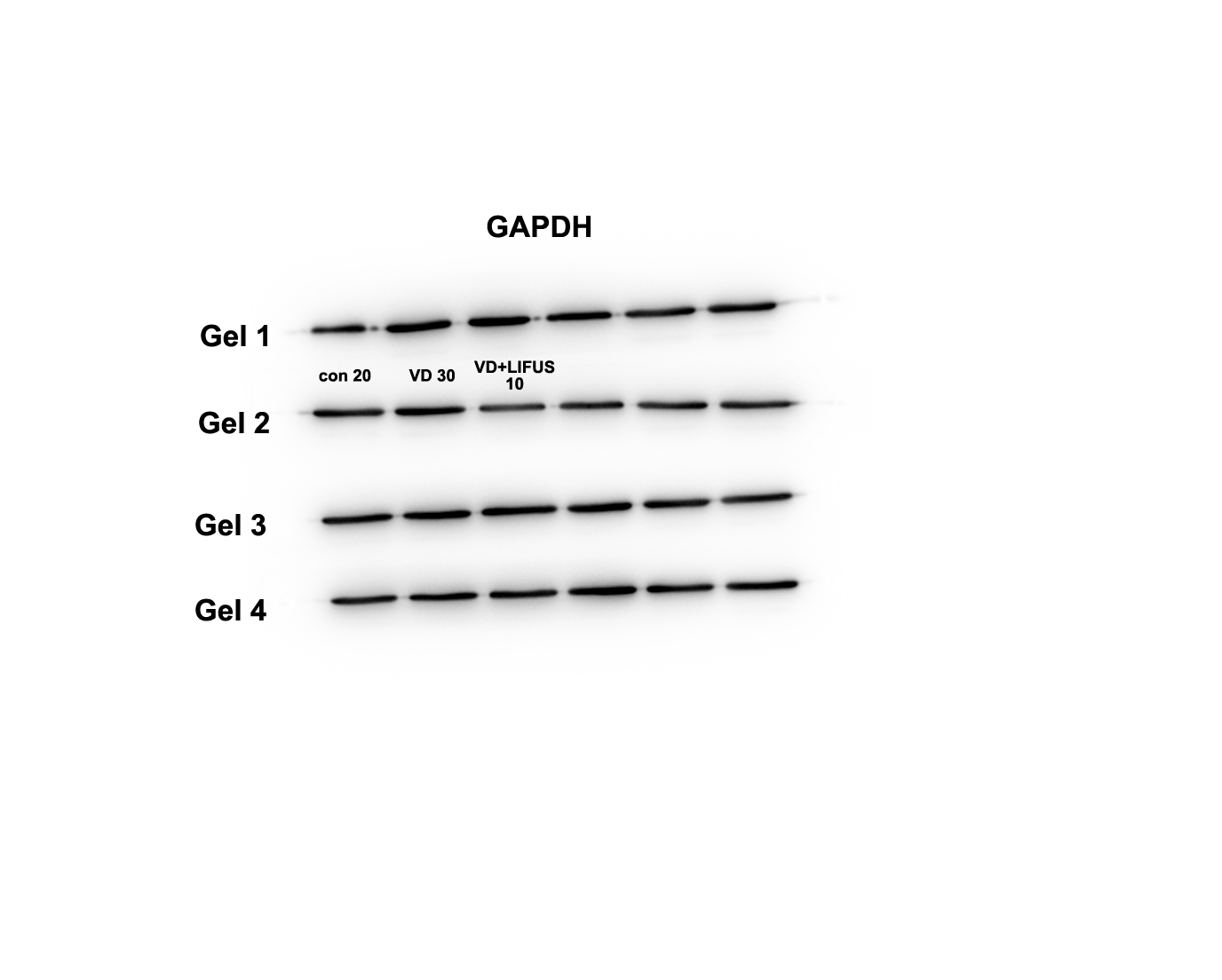

Supplement: Supplementary file 5 [file Data_Sheet_4.ZIP › Original images displayed inmanuscript-Western blot/IL-6/GAPDH.tif]

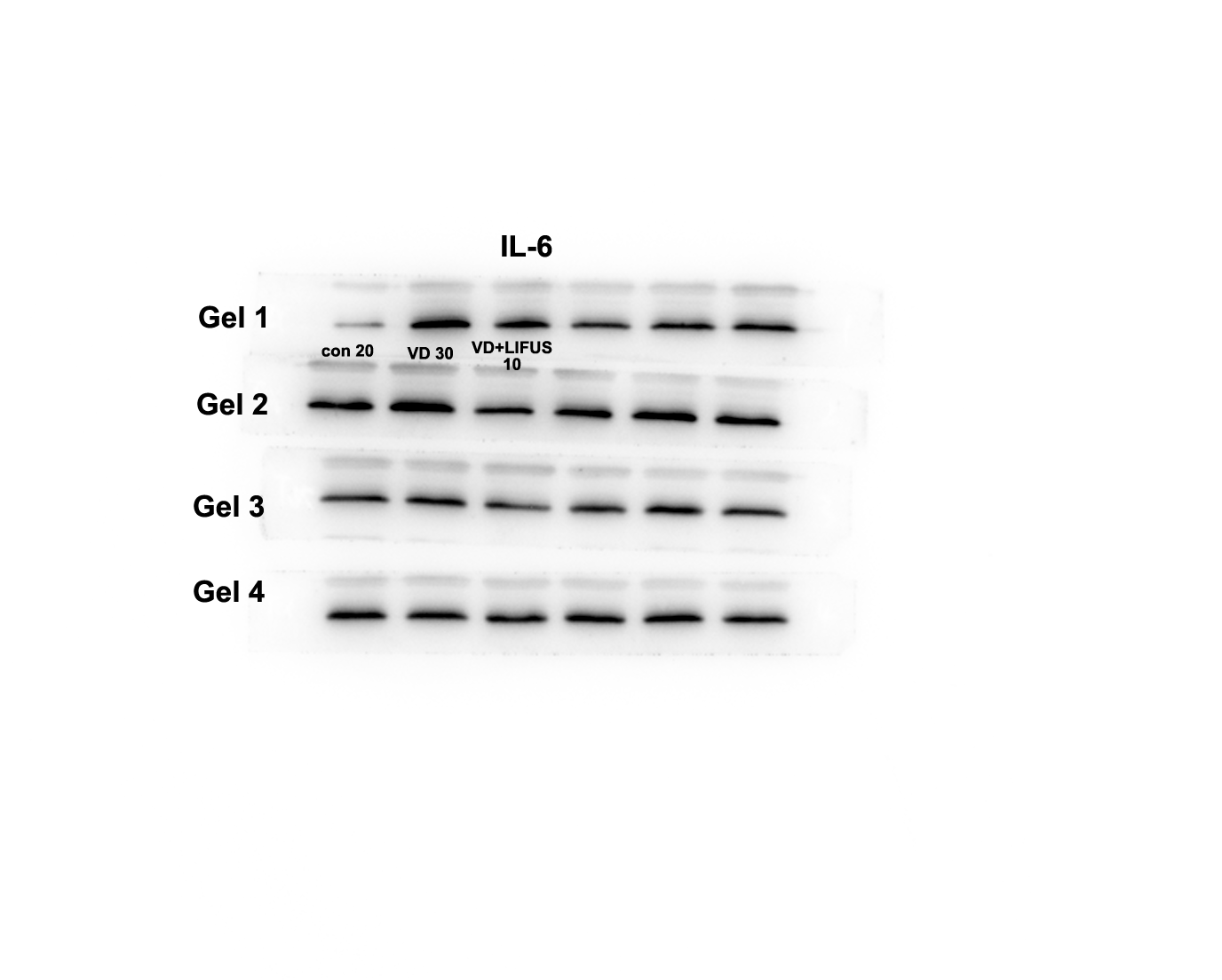

Supplement: Supplementary file 5 [file Data_Sheet_4.ZIP › Original images displayed inmanuscript-Western blot/IL-6/IL6.tif]

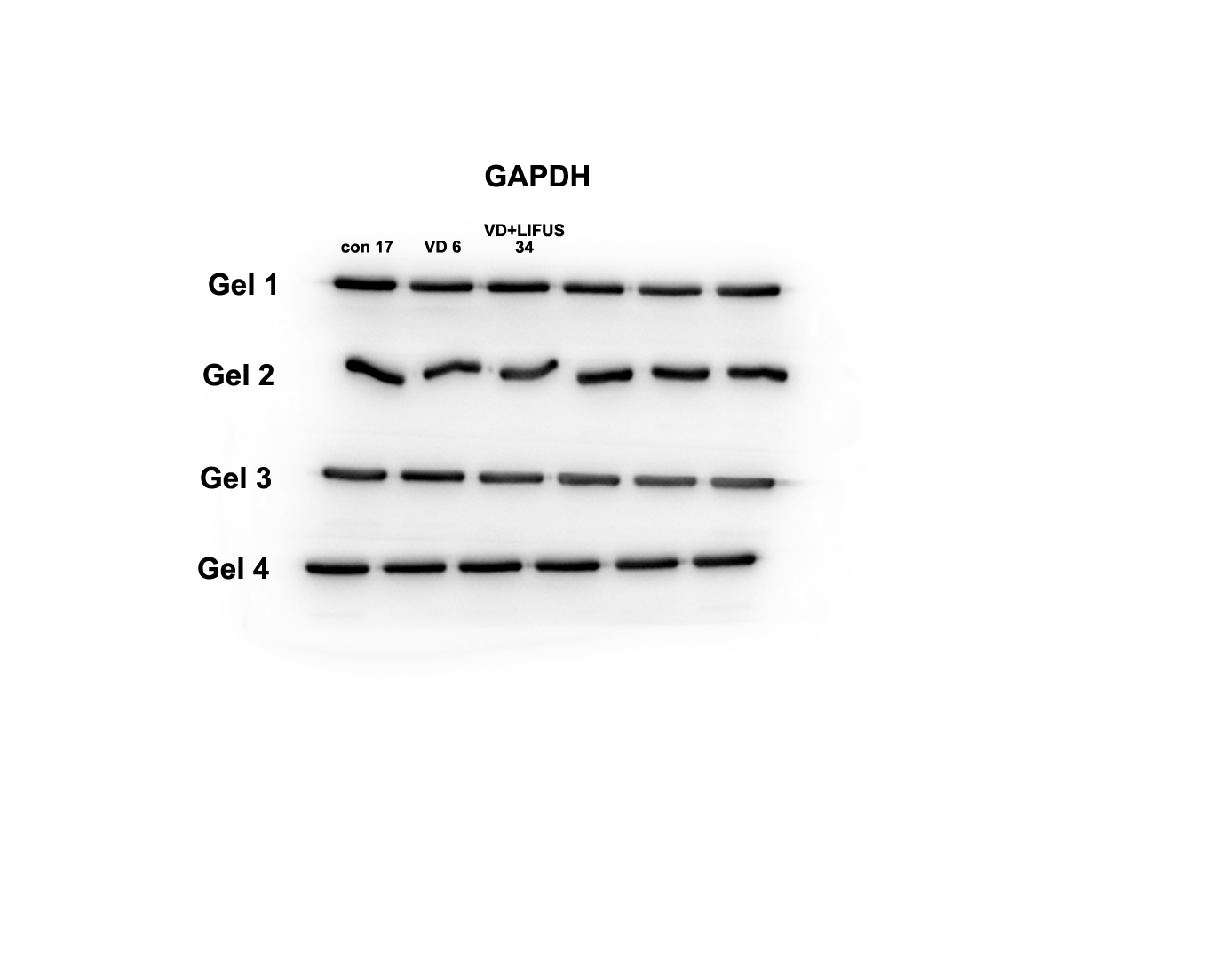

Supplement: Supplementary file 5 [file Data_Sheet_4.ZIP › Original images displayed inmanuscript-Western blot/JNK/GAPDH.tif]

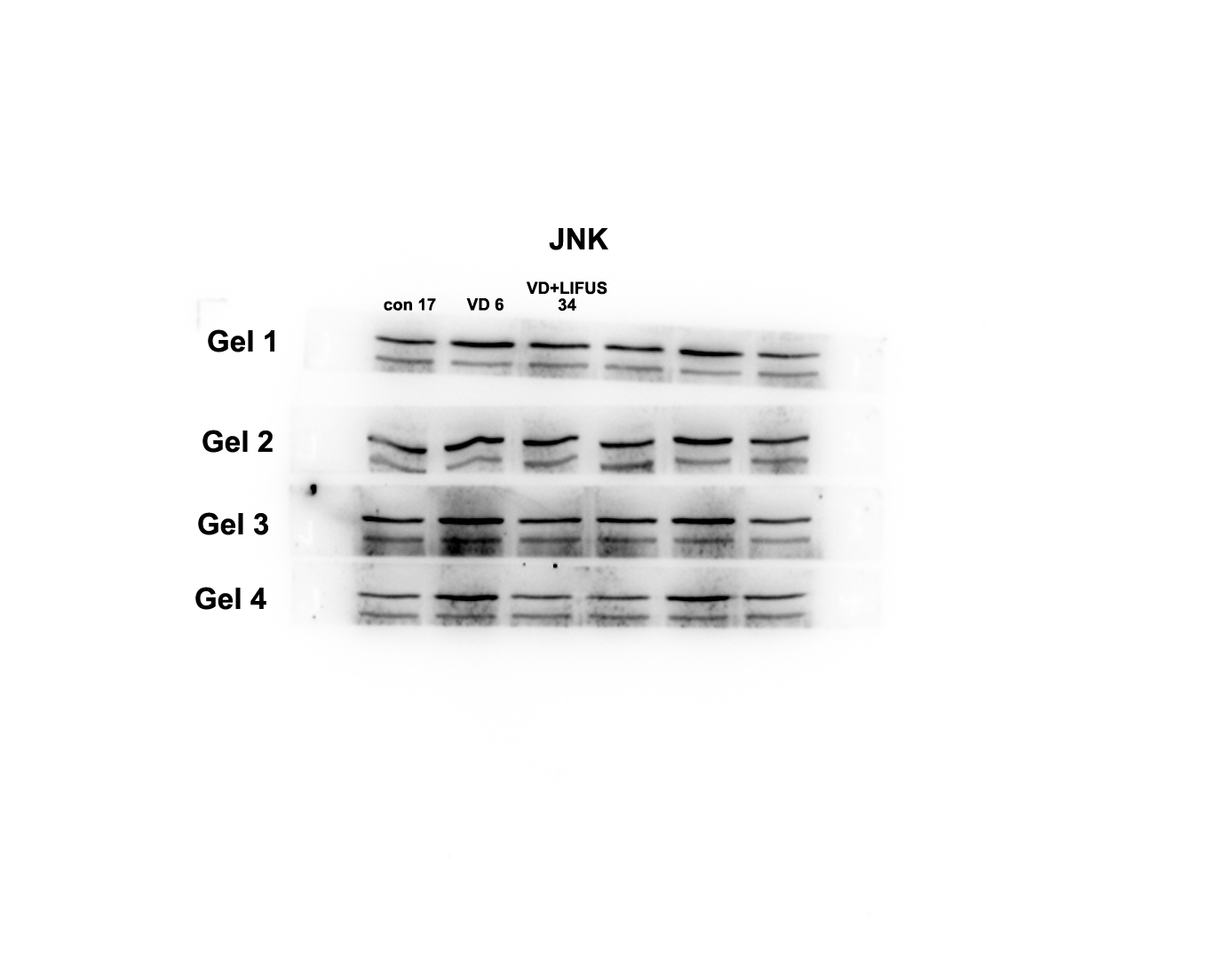

Supplement: Supplementary file 5 [file Data_Sheet_4.ZIP › Original images displayed inmanuscript-Western blot/JNK/JNK.tif]

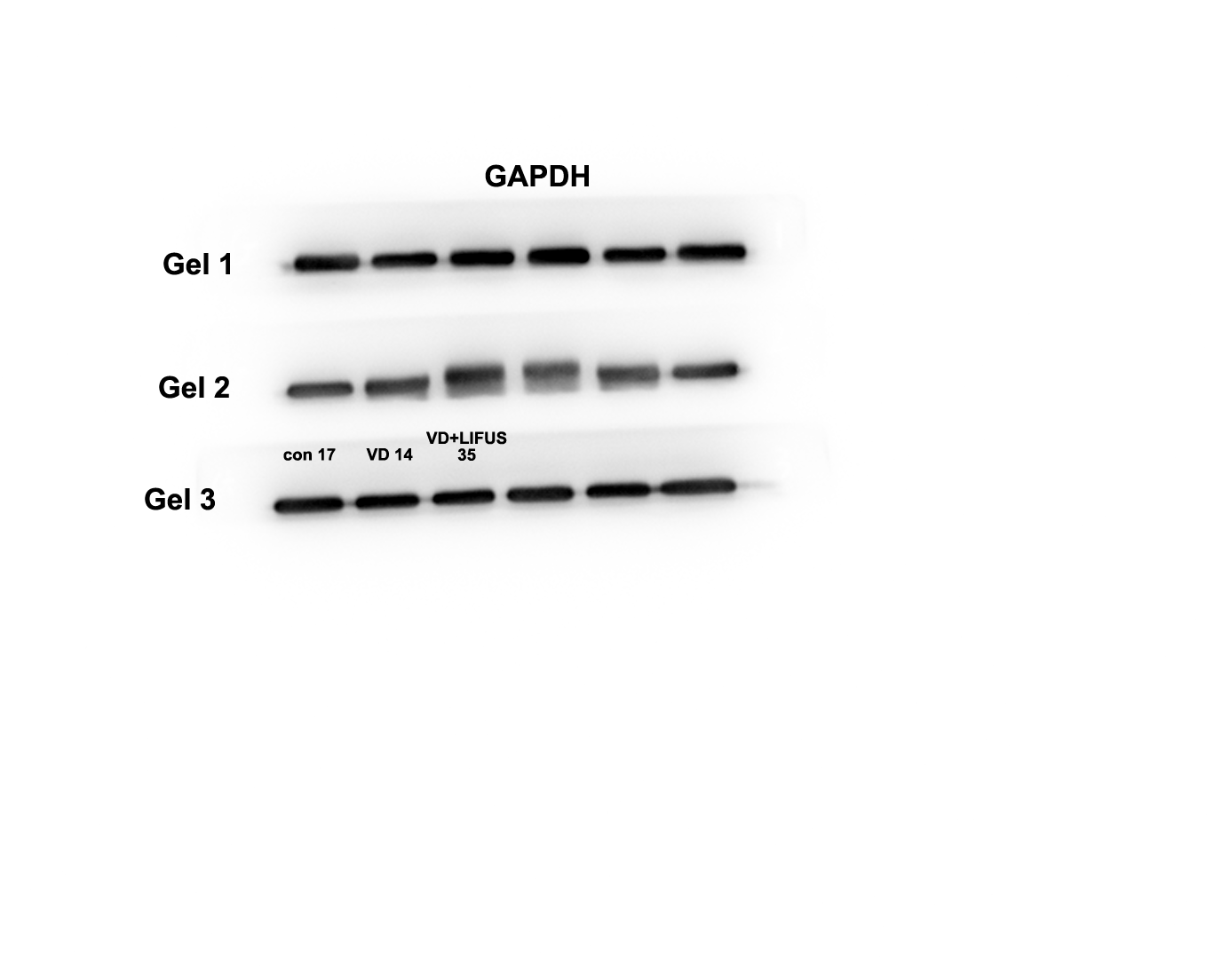

Supplement: Supplementary file 5 [file Data_Sheet_4.ZIP › Original images displayed inmanuscript-Western blot/NF-κB/GAPDH.tif]

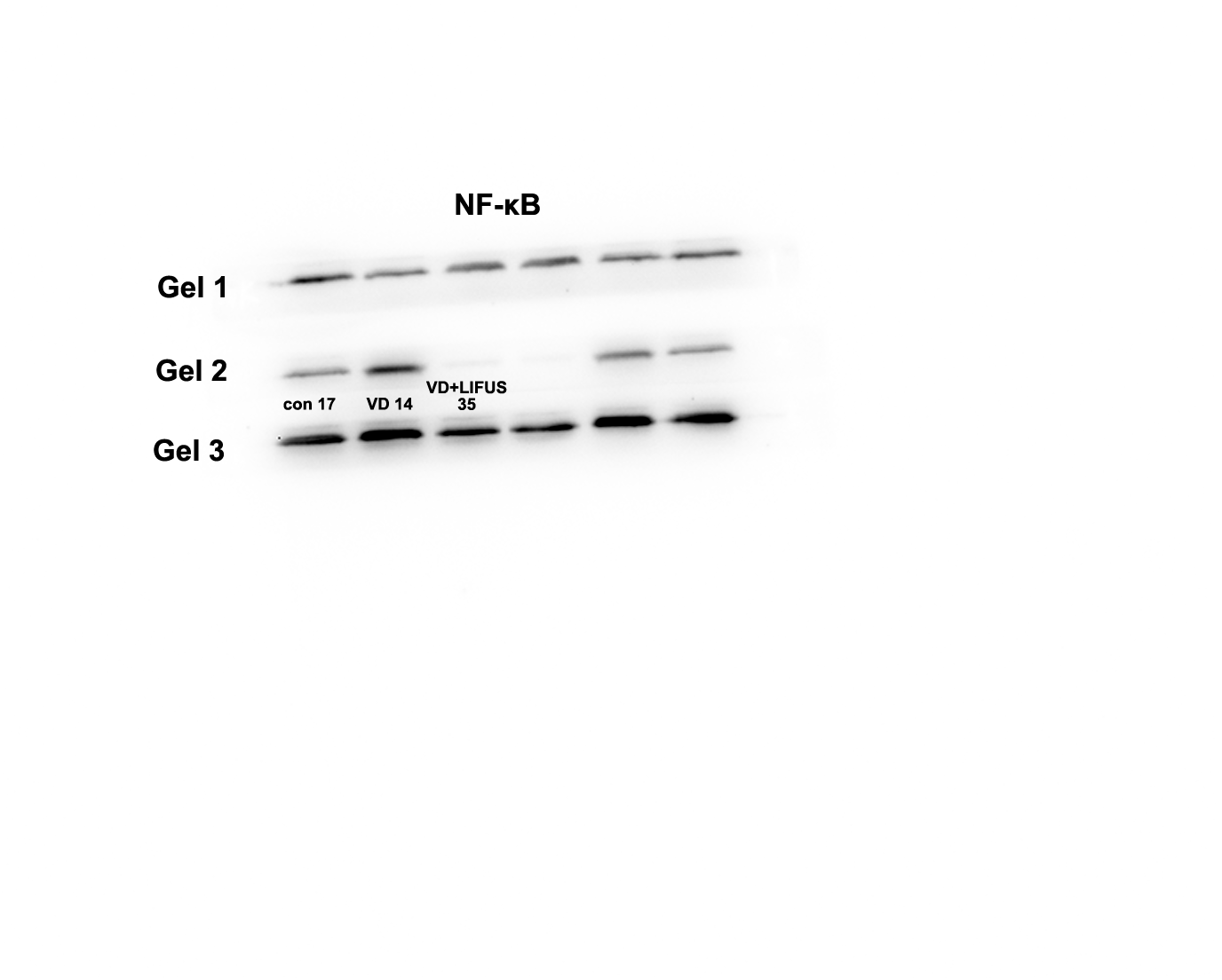

Supplement: Supplementary file 5 [file Data_Sheet_4.ZIP › Original images displayed inmanuscript-Western blot/NF-κB/NFκB.tif]

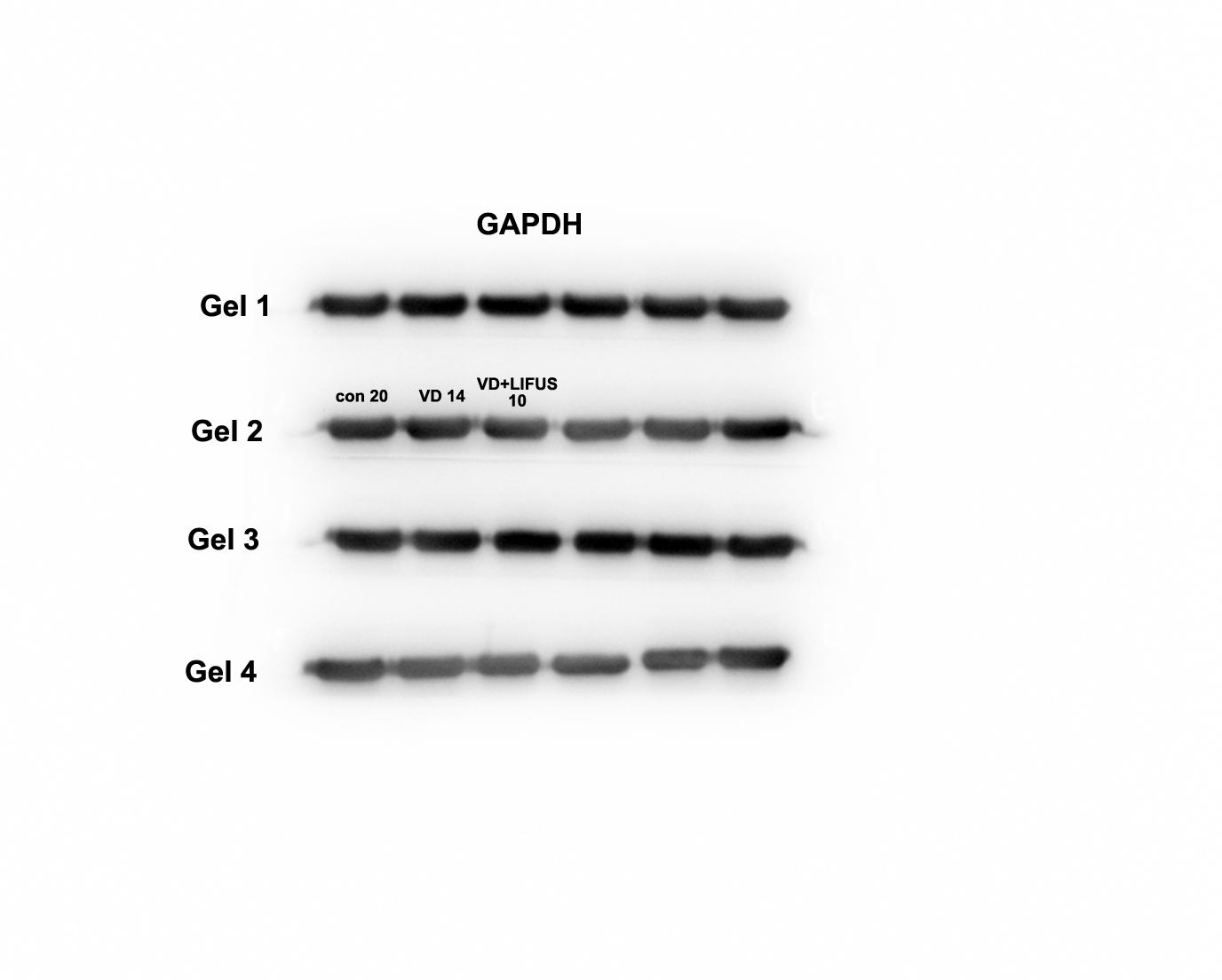

Supplement: Supplementary file 5 [file Data_Sheet_4.ZIP › Original images displayed inmanuscript-Western blot/NR2B/GAPDH.tif]

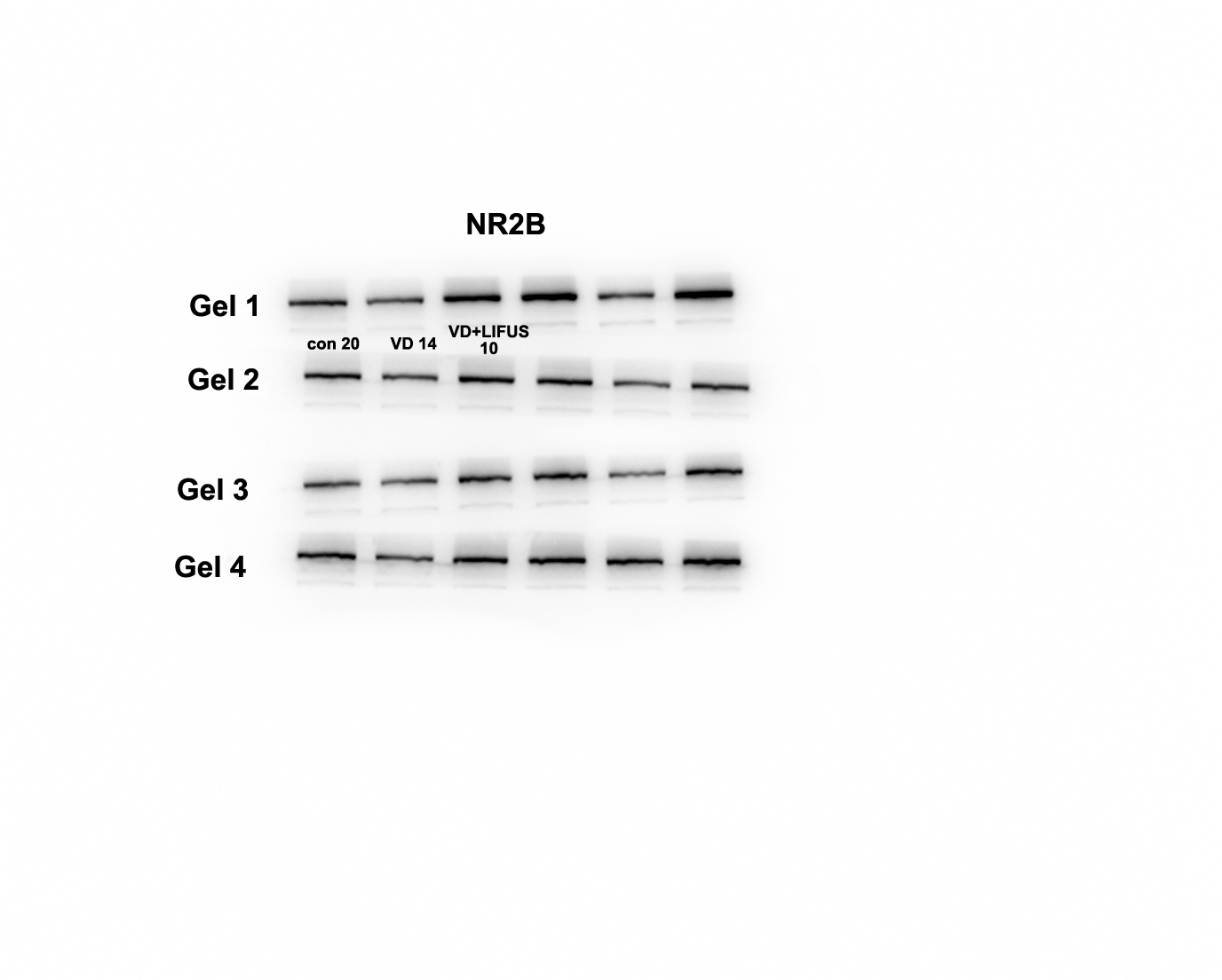

Supplement: Supplementary file 5 [file Data_Sheet_4.ZIP › Original images displayed inmanuscript-Western blot/NR2B/NR2B.tif]

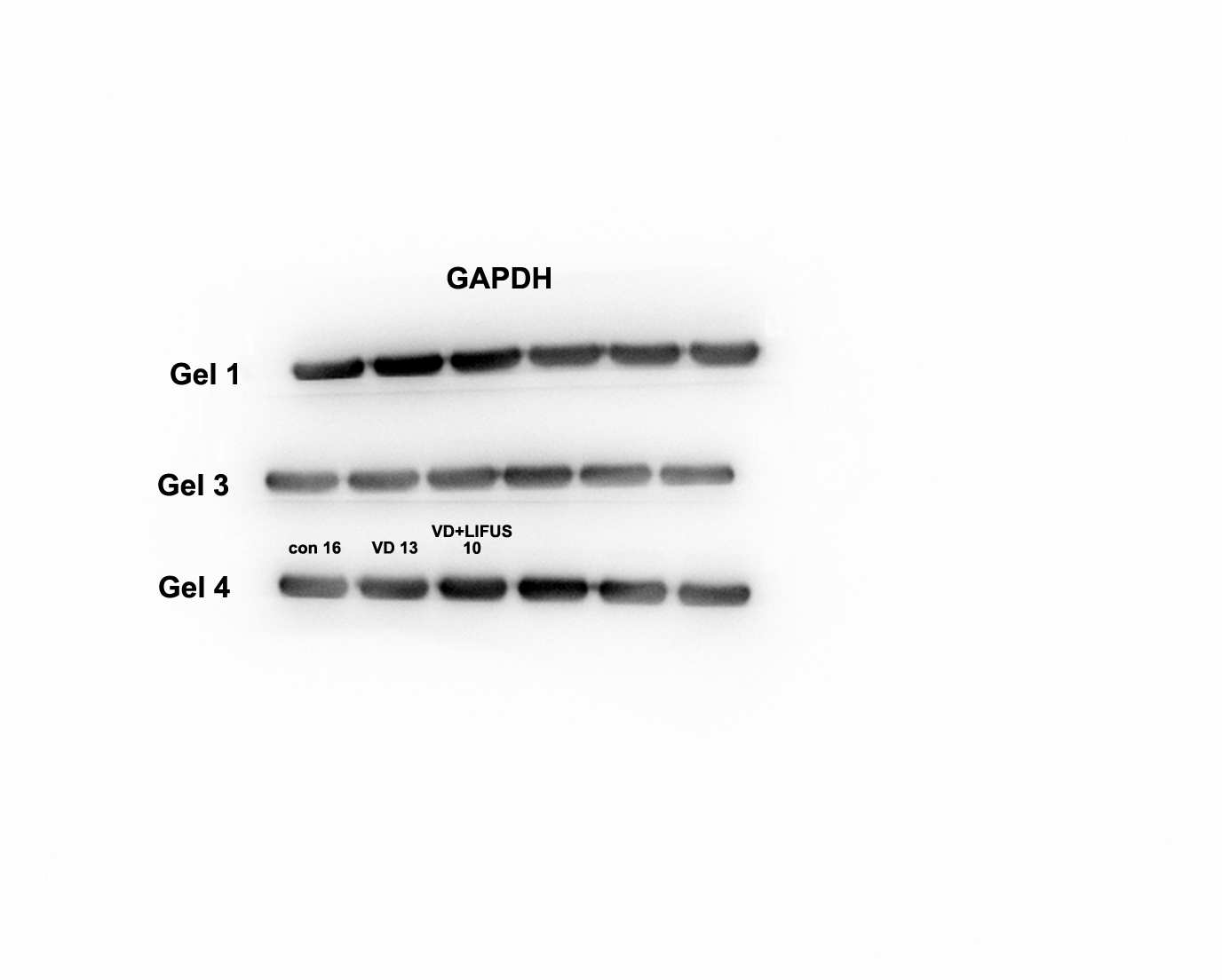

Supplement: Supplementary file 5 [file Data_Sheet_4.ZIP › Original images displayed inmanuscript-Western blot/PSD-95/GADPH.tif]

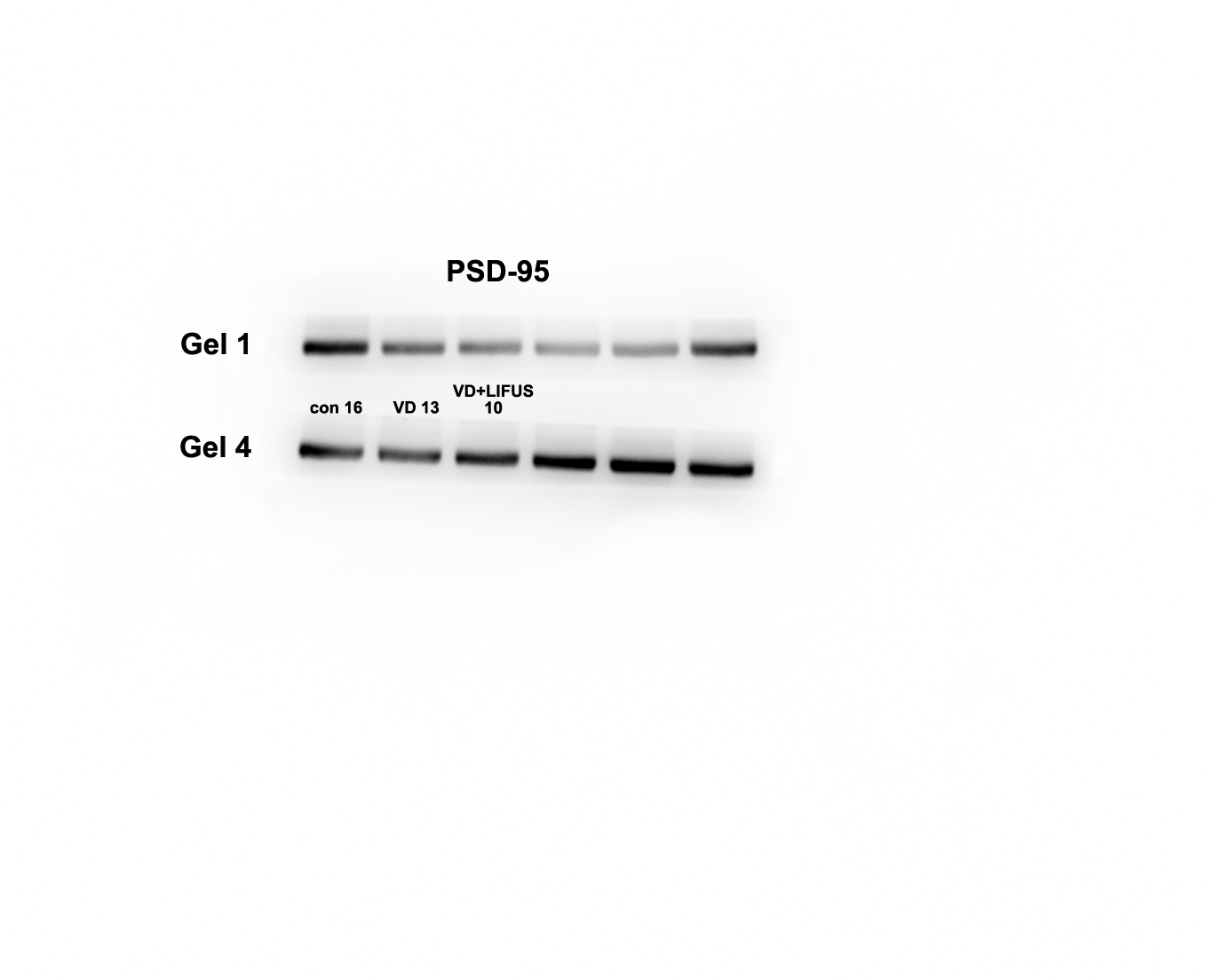

Supplement: Supplementary file 5 [file Data_Sheet_4.ZIP › Original images displayed inmanuscript-Western blot/PSD-95/PSD.tif]

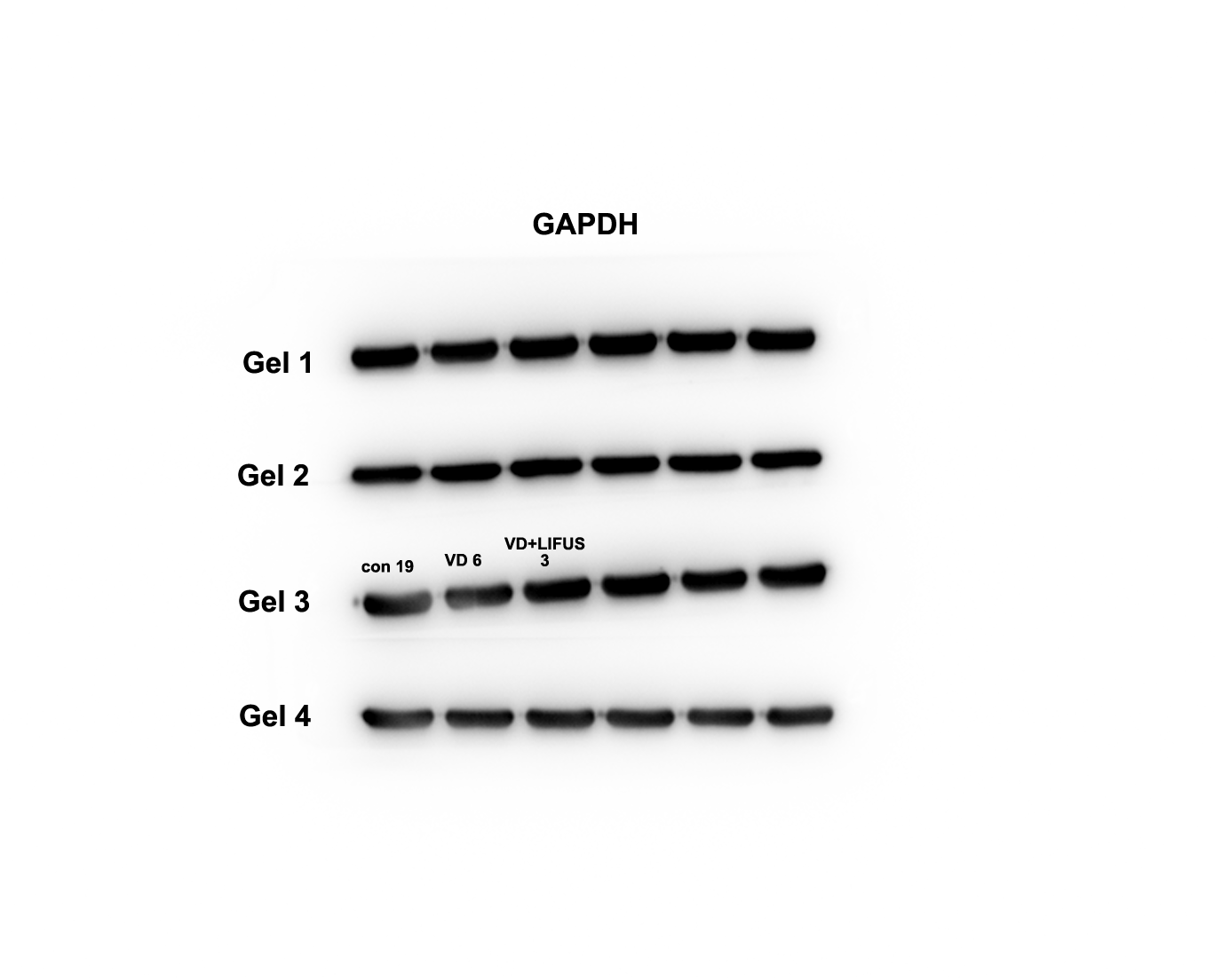

Supplement: Supplementary file 5 [file Data_Sheet_4.ZIP › Original images displayed inmanuscript-Western blot/SYP/GAPDH.tif]

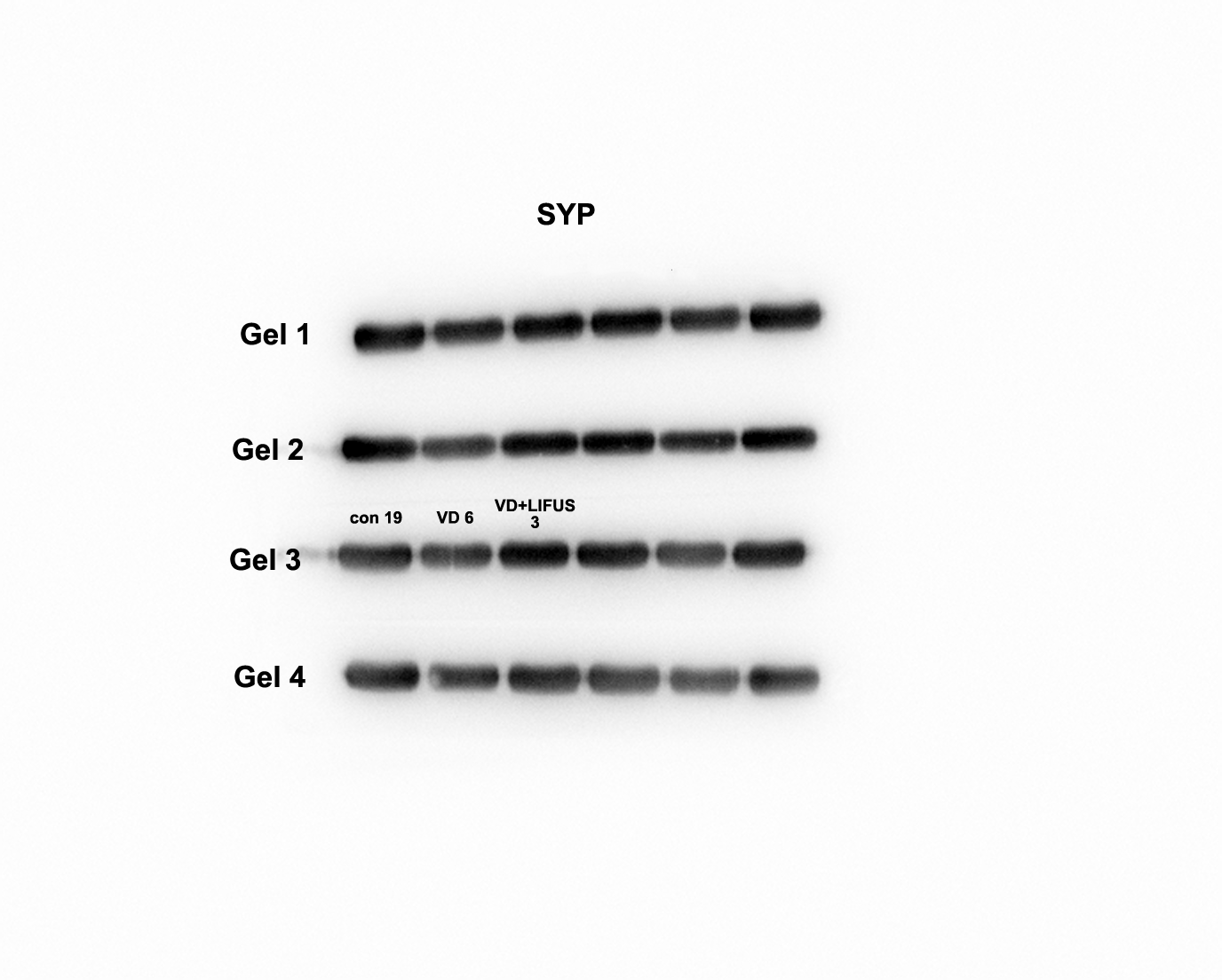

Supplement: Supplementary file 5 [file Data_Sheet_4.ZIP › Original images displayed inmanuscript-Western blot/SYP/SYP.tif]

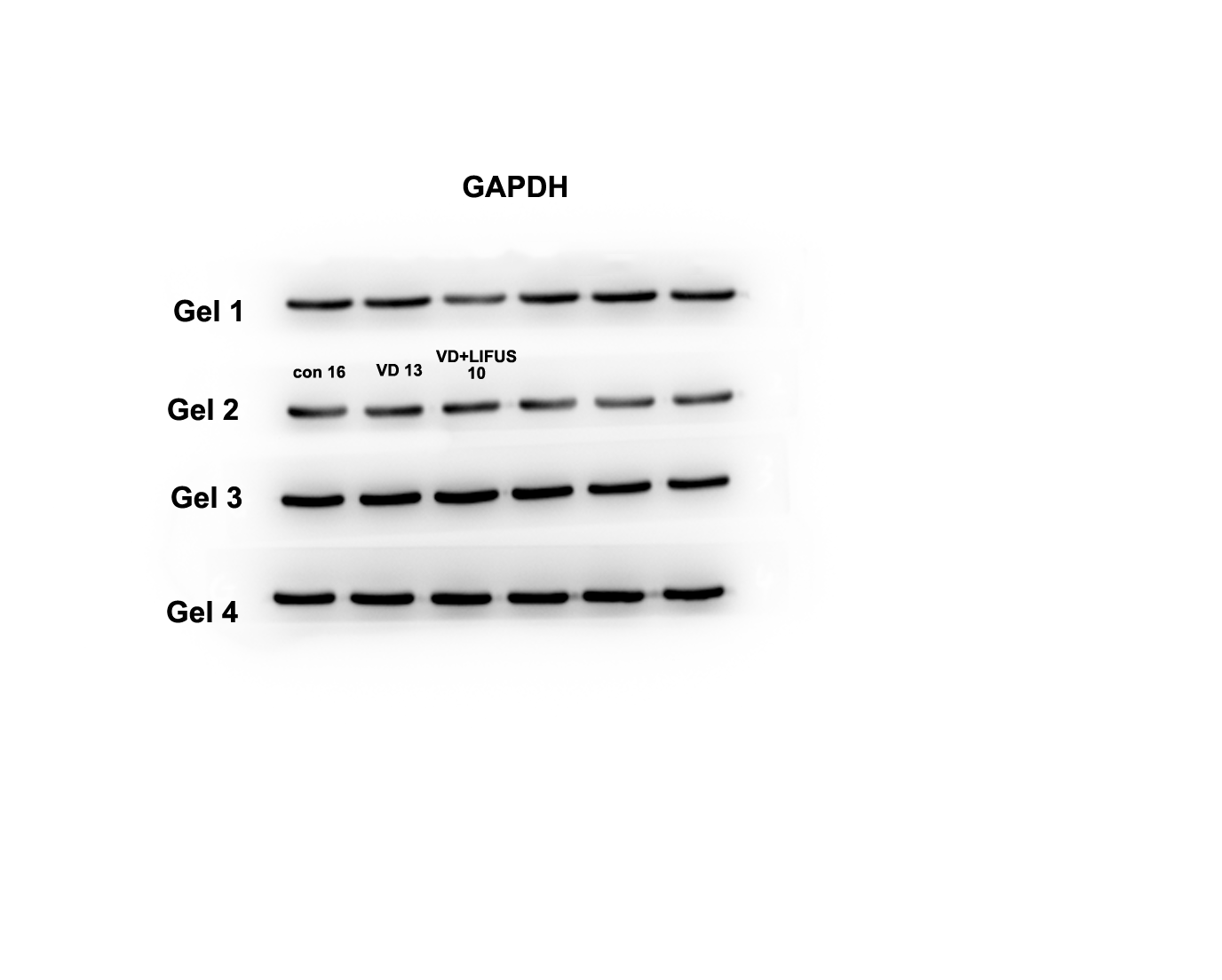

Supplement: Supplementary file 5 [file Data_Sheet_4.ZIP › Original images displayed inmanuscript-Western blot/TLR4/GAPDH.tif]

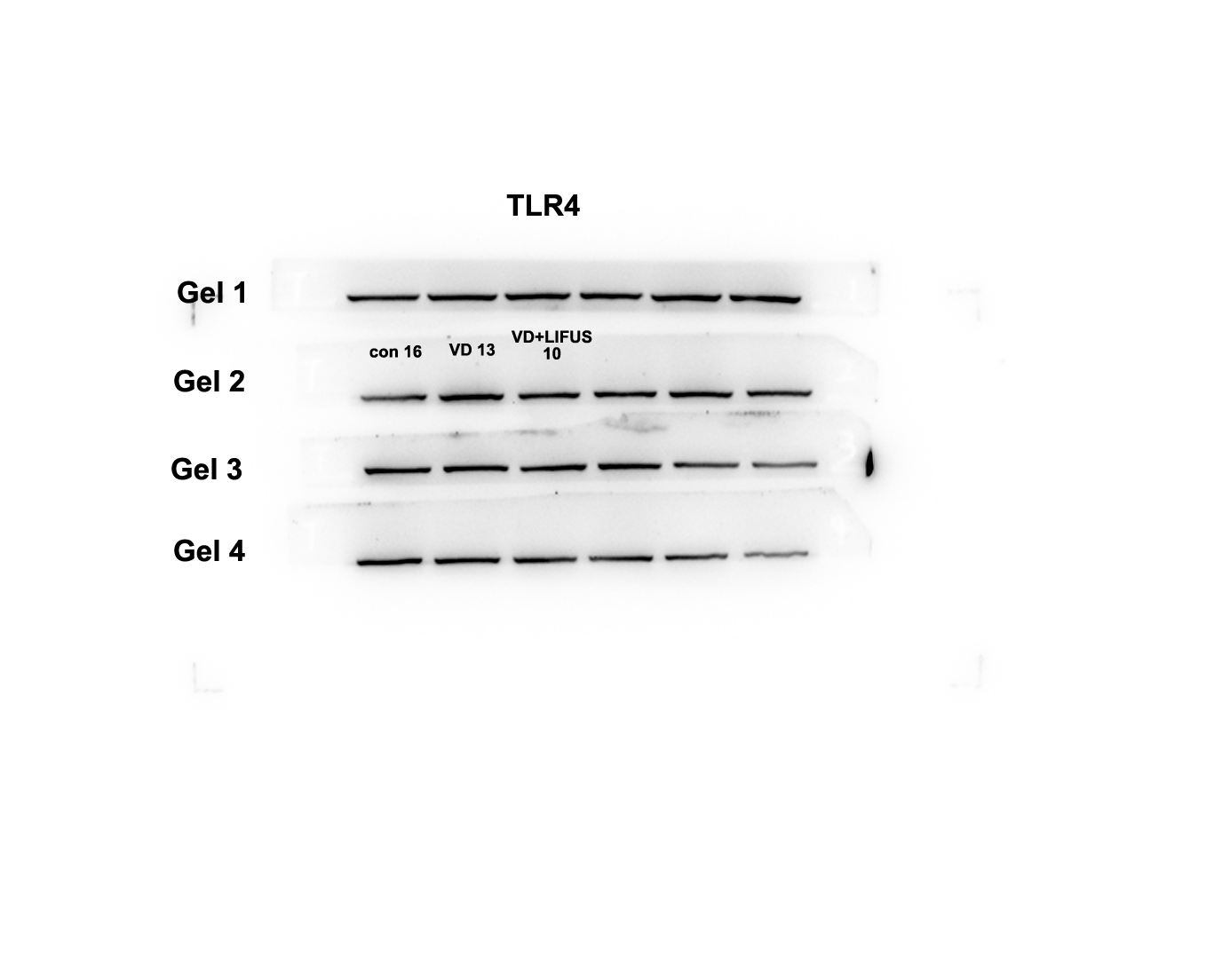

Supplement: Supplementary file 5 [file Data_Sheet_4.ZIP › Original images displayed inmanuscript-Western blot/TLR4/TLR4.tif]

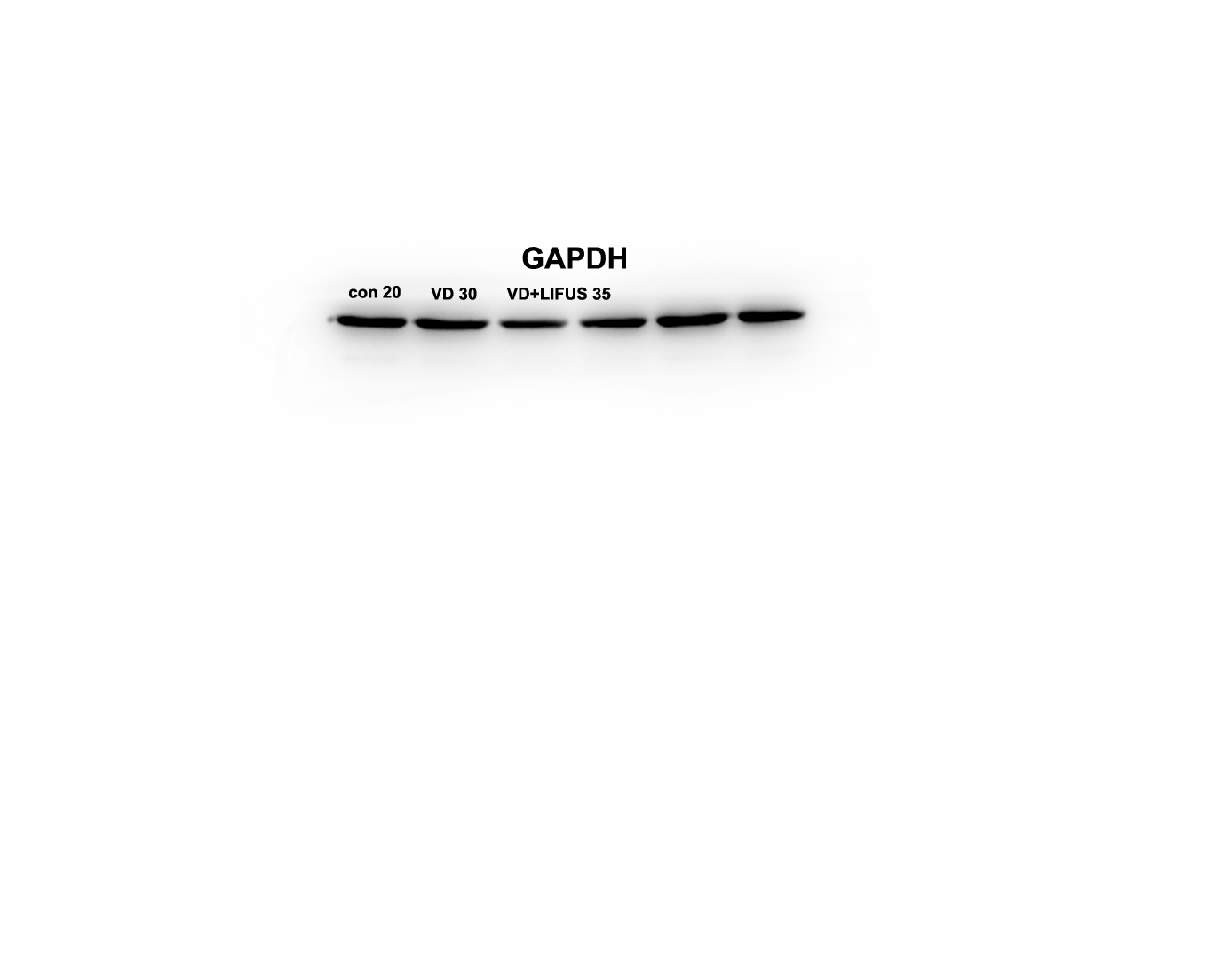

Supplement: Supplementary file 6 [file Image_1.tif]

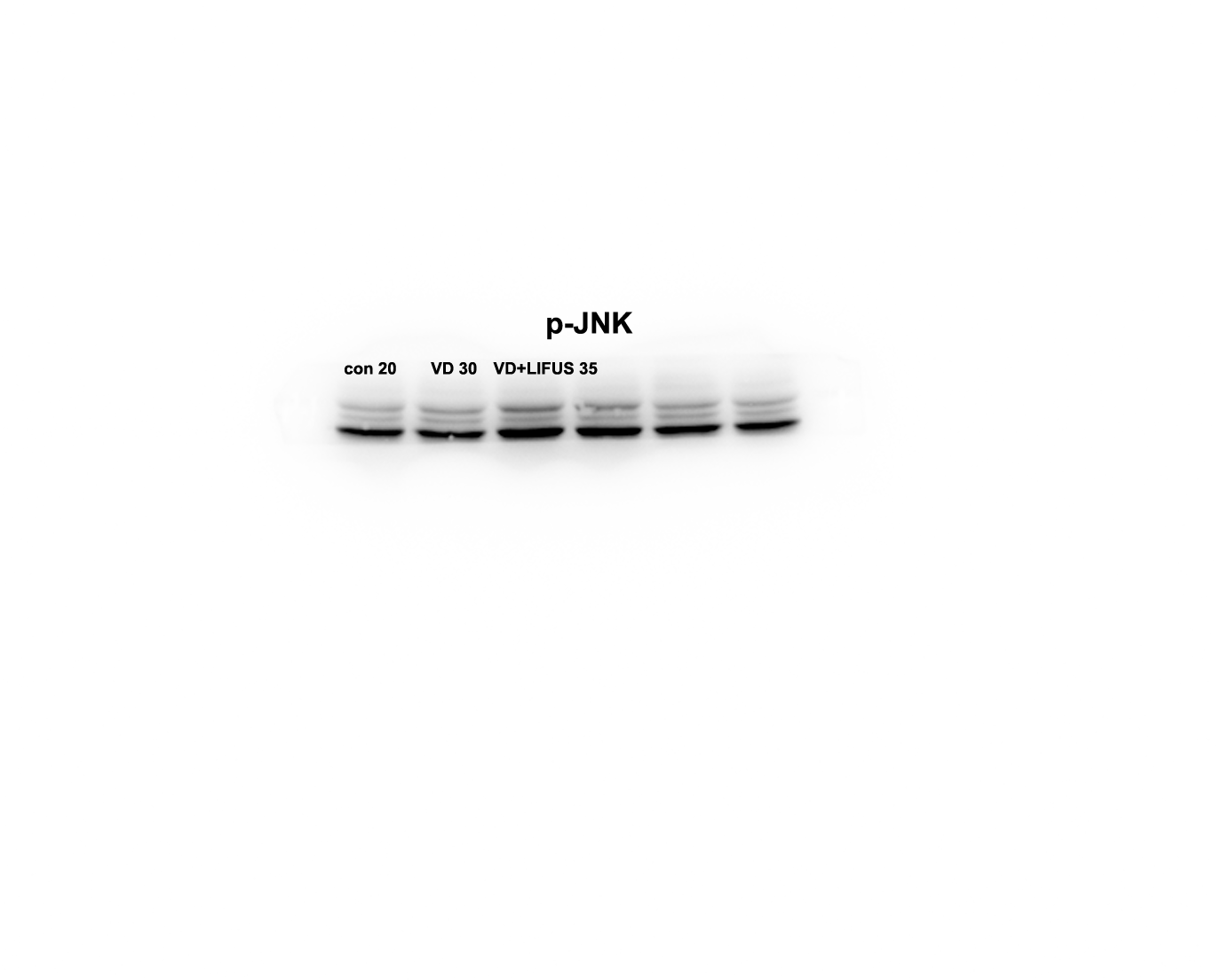

Supplement: Supplementary file 7 [file Image_2.tif]

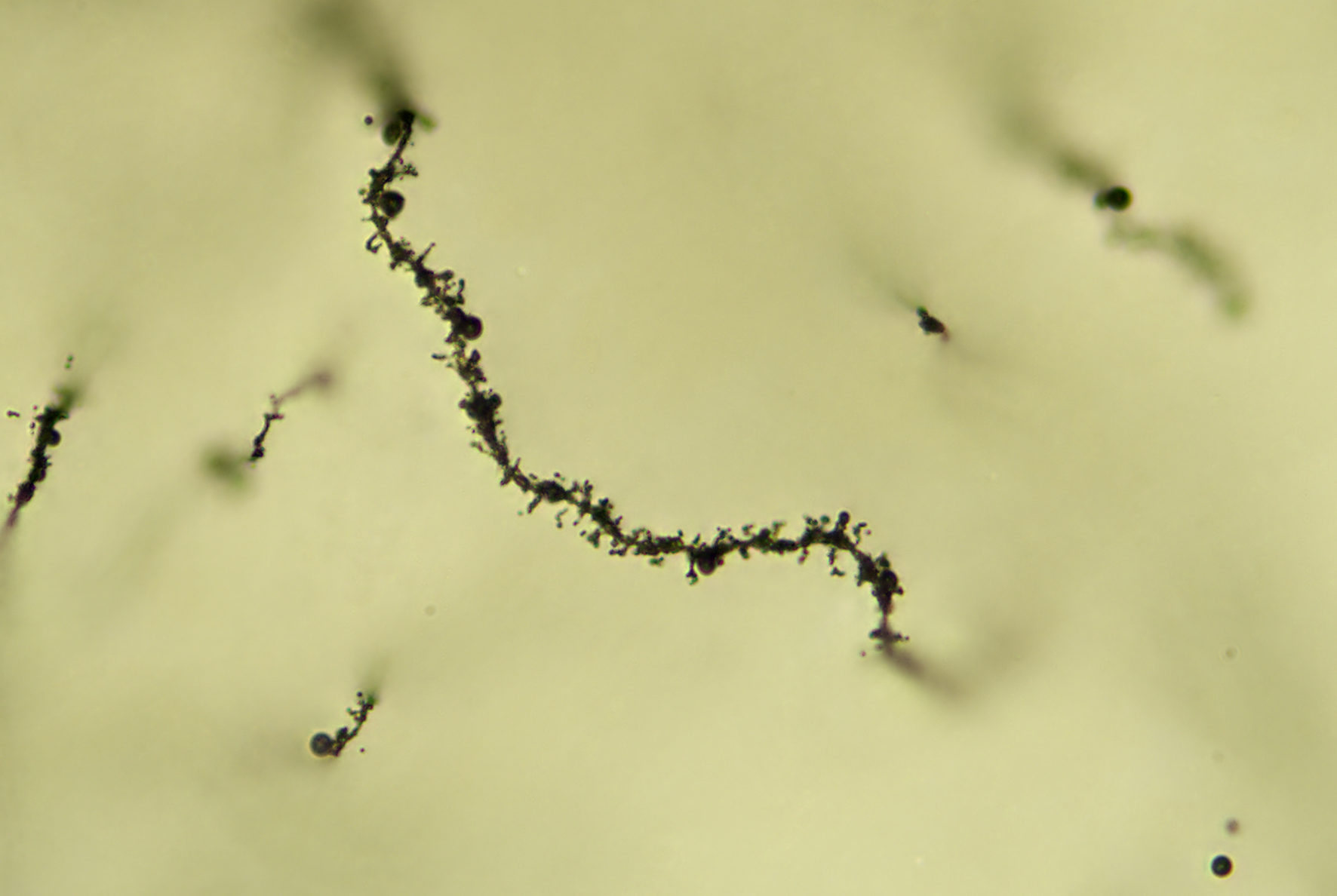

Supplement: Supplementary file 8 [file Image_3.TIF]

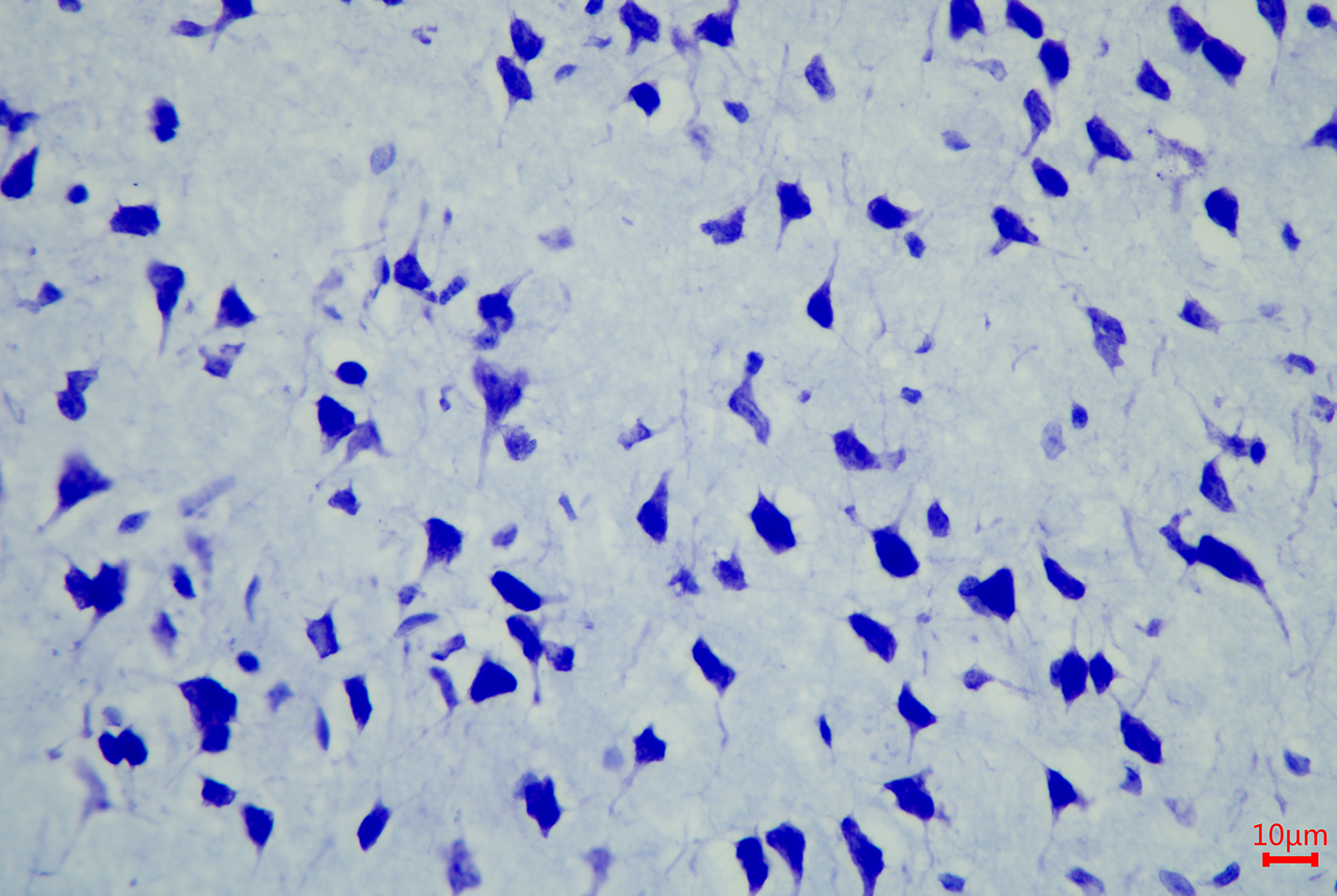

Supplement: Supplementary file 9 [file Image_4.TIF]

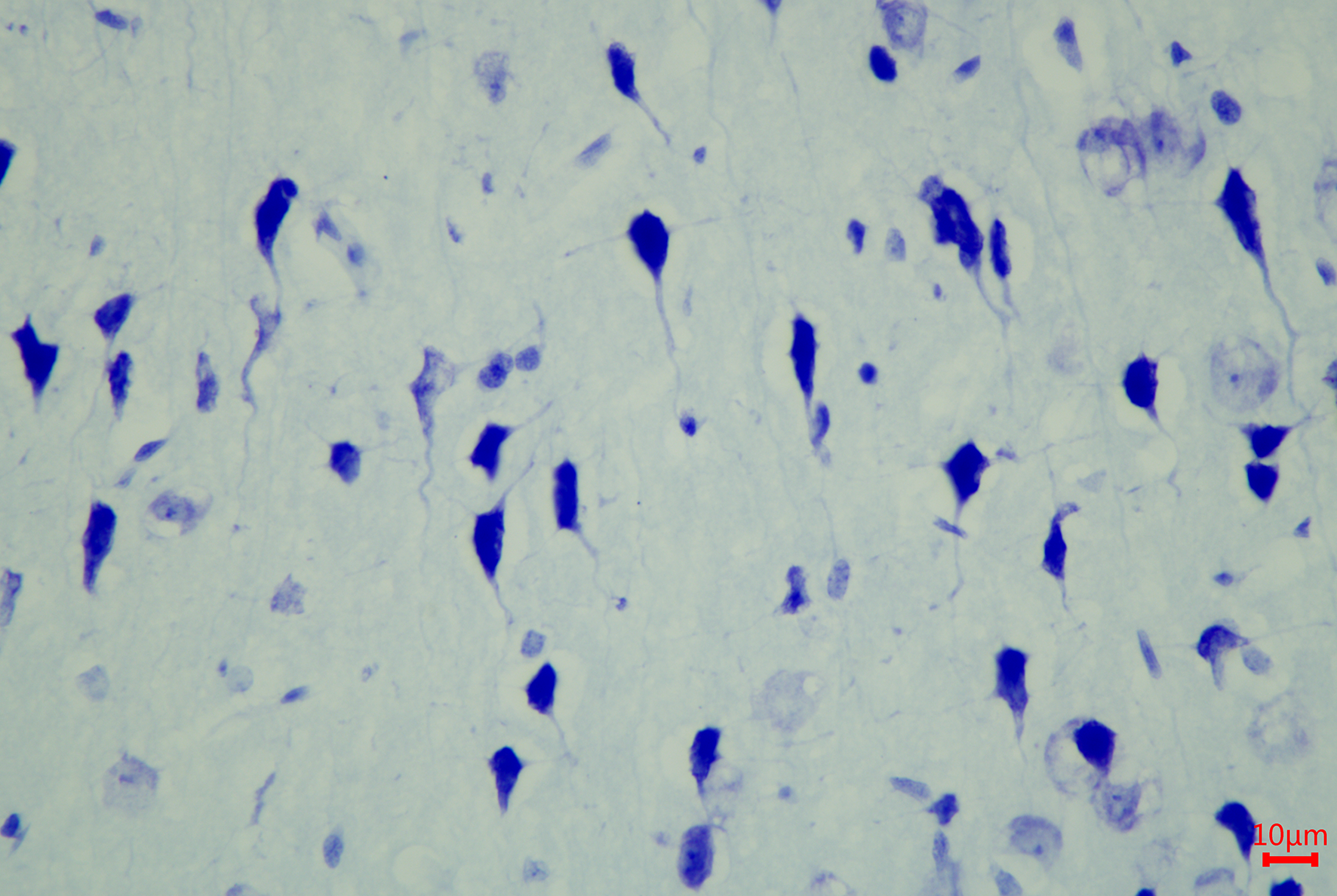

Supplement: Supplementary file 10 [file Image_5.TIF]

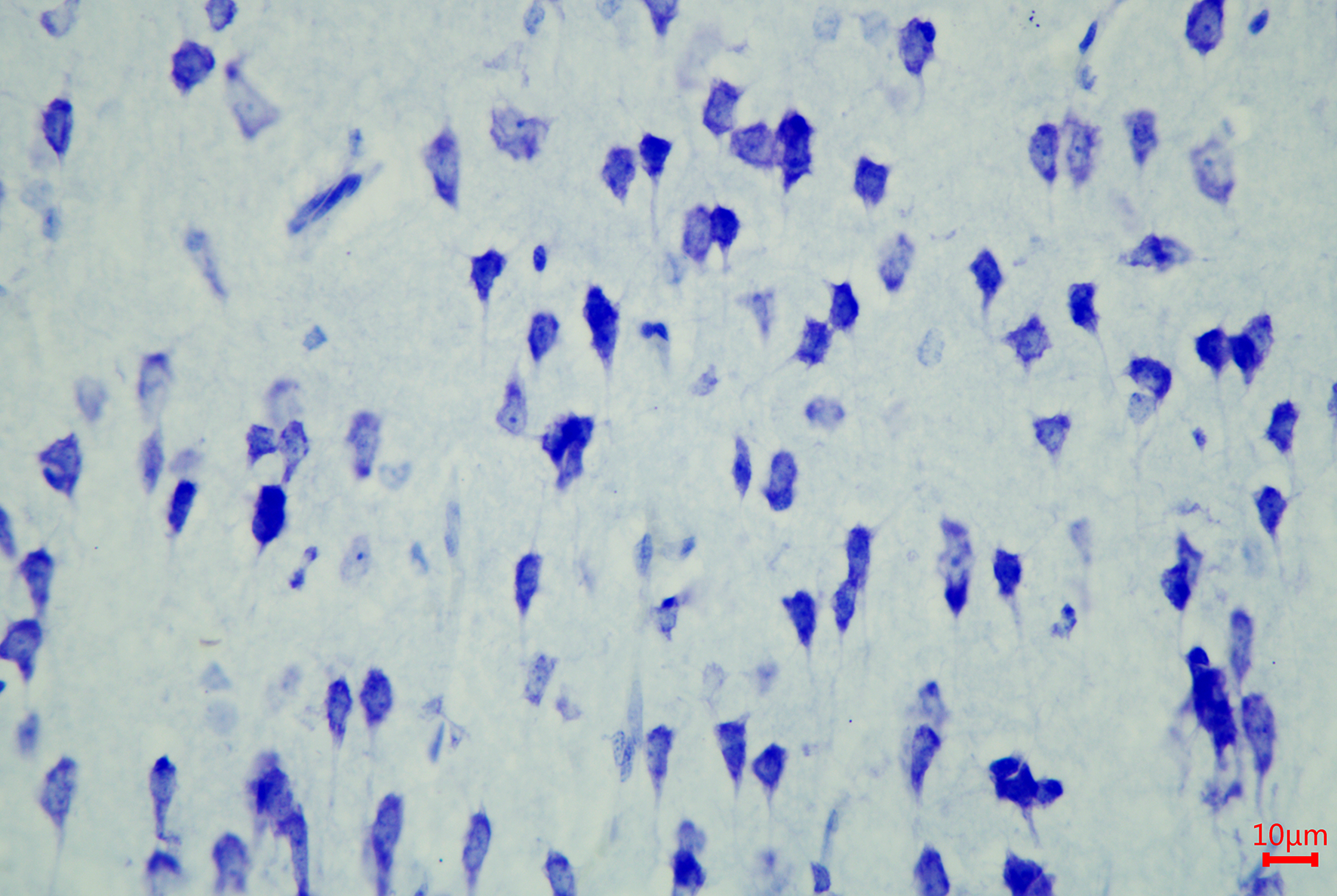

Supplement: Supplementary file 11 [file Image_6.TIF]
